# Whole genome sequencing-based multi-locus association mapping for kernel iron, zinc and protein content in groundnut

**DOI:** 10.1038/s41598-026-57575-2

**Published:** 2026-06-13

**Authors:** U. Nikhil Sagar, Sejal Parmar, Sunil S. Gangurde, Vinay Sharma, Arun K. Pandey, D. Khaja Mohinuddin, Namita Dube, Ramesh S. Bhat, K. John, Muga D. Sreevalli, P. Sandhya Rani, Kuldeep Singh, Rajeev K. Varshney, Manish K. Pandey

**Affiliations:** 1 Center for Pre-Breeding Research (CPBR), International Crops Research Institute for the Semi-Arid Tropics (ICRISAT), Hyderabad, Telangana 502324 India; 2 Acharya N.G. Ranga Agricultural University, Guntur, 522034 India; 3 Discipline of Biotechnology, University of Agricultural Sciences, Dharwad, 580005 India; 4 Centre for Crop and Food Innovation, WA State Agricultural Biotechnology Centre, Murdoch University, Murdoch, Australia

**Keywords:** Candidate genes, Fe and Zn homeostasis pathway, KASP markers, Marker trait associations, Nutritional quality, Biotechnology, Computational biology and bioinformatics, Genetics, Molecular biology, Plant sciences

## Abstract

Malnutrition is a major global challenge, especially in the developing regions, where improving the nutritional content of staple crops is an important step towards alleviating hidden hunger. Groundnut, a nutrient rich legume, contains several essential nutrients, high protein, essential amino-acids and vitamins required for human health. In this study, multi-season phenotyping data for kernel iron (Fe), zinc (Zn) and protein content (PC), along with whole genome re-sequencing (WGRS) data from a mini-core collection, were used to perform genome-wide association study (GWAS) analysis. Phenotypic variability analysis revealed a large variation in Fe (7.6 – 42.8 ppm), Zn (10.9 – 62.4 ppm) and PC (12.7 – 33.6%). GWAS analysis identified a total of 15 marker-trait associations (MTAs) and 28 candidate genes for pooled season data, and 44 MTAs and 62 candidate genes for individual seasons. Key candidate genes like *MYB transcription factor* (*Arahy.QI0PHV*, *Arahy.1I6ZSS*), *Zn finger MYM type protein*, *RING finger MYM type protein* (*Arahy.7P97F6*, *Arahy.9R964H*, *Arahy.I3B88T*) and *NAC domain protein* (*Arahy.LV3APC*), were found to be associated with the Fe and Zn homeostasis pathway. In addition, genes related to protein homeostasis, such as *Protein kinase family protein* (*Arahy.4D7KBI*), and *E3 ubiquitin-protein ligase* (*Arahy.PE3CF6*), were identified within significant MTA regions. These findings provide basis for the detection and characterization of potential candidate genes associated with nutritional quality traits. Haplo-pheno analysis revealed that accessions with superior haplotypes for kernel Fe and Zn content were predominantly found in the Spanish Bunch/Valencia Bunch types, whereas the accessions with inferior haplotypes were more common in the Virginia Bunch/Virginia Runner types. Single nucleotide polymorphism (SNP)-based KASP (Kompetitive Allele Specific Polymerase Chain Reaction) markers for 9 MTAs were designed and validated for Fe and Zn. Among these, three markers (snpAH00636 and snpAH00641 for Fe, and snpAH00644 for Zn) showed polymorphism, and could be utilized in genomics-assisted breeding to develop nutrient-rich groundnut varieties.

**Supplementary Information:**

The online version contains supplementary material available at https://doi.org/10.1038/s41598-026-57575-2.

## Introduction

Groundnut (*Arachis hypogaea* L.), often referred to as the "King of oilseeds", is valued for its high oil content, nutritional value, and economic importance. It is also referred to as peanut, earthnut, monkey nut, wonder nut, and goobernut^1^. Globally, it is the fourth-most important source of vegetable protein. Groundnut is a self-pollinated, segmental allotetraploid crop with minimal molecular-level variation^2^. Taxonomically, it belongs to the family Leguminosae and sub-family Papilionaceae^3^. Originating in South America, it is now widely cultivated across tropical, sub-tropical, and semi-arid regions of the world. Currently, groundnut is grown on over 32.7 million hectares across more than 100 countries, producing approximately 53.9 million tonnes, with an average productivity of 1.64 tonnes ha^−1^^4^. China and India are the leading producers, contributing 18.38 million tonnes (34.3%) and 10.13 million tonnes (17.4%) respectively, followed by Nigeria- 4.28 million tonnes (8%), USA- 2.53 million tonnes (5%) and Sudan- 2.50 million tonnes (5%), Among these, the USA records the highest productivity of 4469.26 kg ha^−1^^4^.

Groundnut, as a nutrient rich legume crop, contains upto 28% protein, 50% oil, and 20% carbohydrates^5^. Moreover, it is also an important source of monounsaturated and polyunsaturated fatty acids, bioactive compounds such as polyphenols, flavonoids, isoflavones, and several B-complex vitamins, and antioxidants, including resveratrol and p-coumaric acid. Raw groundnut contains 4.58 mg of Fe, 3.27 mg of Zn and 26 g of PC per 100 g. Fe and Zn are important micronutrients required for human growth and development, and also serve as cofactors for proteins like haemoglobin and cytochromes^5^.

Malnutrition, or "hidden hunger," particularly Fe and Zn deficiencies, affects nearly three billion people worldwide^6^, with a significant proportion of women and children in India suffering from anemia. Due to its nutritional richness, groundnut and its products can be promoted as nutrient-dense foods to address protein, energy, and micronutrient deficiencies in vulnerable populations^7^. For example, ready-to-use therapeutic foods (RUTFs) such as Plumpy’Nut®, derived from groundnut, have been highly effective in saving the lives of malnourished children in several regions^8^. Recent advances in genomic resources have opened new avenues for enhancing the nutritional profile of groundnut. These resources include whole-genome resequencing (WGRS), skim sequencing, mid-density platforms, genotyping-by-sequencing (GBS), restriction site-associated sequencing (RADSeq), double- digest restriction site-associated sequencing (ddRADSeq), and specific length amplified fragment sequencing (SLAF-seq); reference genomes for diploid wild progenitors (*A. duranensis* and *A. ipaensis*) and cultivated tetraploid genomes (*A. hypogaea* subsp*. hypogaea* and *A. hypogaea* subsp. *fastigiata*); and diagnostic markers such as simple sequence repeats (SSRs), insertions and deletions (InDels), and SNPs^9^. Genomic tools successfully contributed in the development of several varieties with traits of interest, such as high oleic acid, resistance to rust and late leaf spot (LLS)^10^. High and mid-density genotyping arrays/assays have further enabled the construction of genetic maps, identification of genomic regions and candidate genes via GWAS, and the development of diagnostic markers to accelerate breeding programs^5^. These tools also facilitate various applications, such as assessing genetic purity in seed systems and gene bank germplasm, foreground and background selection in backcross breeding, trait mapping, and genomic selection in groundnut^10^.

Groundnut is consumed as a part of the human diet, making it one of the most nutrient-dense food crops^11^. Despite its nutritional value, further enrichment of micronutrients like Fe and Zn,  and PC are essential for improving human health, particularly in developing and underdeveloped regions^12^. Integrating genomic tools with robust phenotyping approaches in groundnut breeding programs offers promising opportunities to enhance nutritional quality. Groundnut biofortification efforts can be further strengthened by identifying and characterizing candidate genes associated with nutrient accumulation.

Several key candidate genes encoding transcription factors such as *NAC*, *MYB* were reported to regulate seed and pod development, plant growth, and photosynthesis, under Fe deficiency conditions^13^. Proteins encoded by *MYB transcription factor*, *RING finger protein*, and *NAC transcription factor* were reported to have a key role in Fe and Zn homeostasis pathways in seeds^5^. Earlier studies also identified homologs such as *IRT1*, *FIT1*, *bZIP23*, and *OPT3*, in groundnut and other legumes, which are involved in Fe transport from leaves to roots^14^. Previous studies in cereals identified several genes or gene families including *YSL*, *NRAMP*, *ZIP*, *Heavy metal ATPase*, *cation diffusion facilitator family*, *nicotianamine synthase*, and *nicotianamine aminotransferase*, responsible for Fe and Zn homeostasis^15^.

Nowadays, GWAS has proven to be highly effective for mapping complex traits across a wide range of populations, including natural and multi-parent breeding populations with varying genetic bases. The main challenge of GWAS is to minimize false positives caused by population structure and family relatedness^16^. Traditional models such as Mixed Linear Model (MLM) attempts to reduce this challenge by including these two factors as covariates^17^, but cause overfitting, resulting in false positives and missing key loci. Multi-locus models offer an advantage by capturing additional loci^18^. For example, Multiple Loci Mixed Linear Model (MLMM) incorporates related markers as cofactors in the mixed model by stepwise regression^19^. More recently, Bayesian-information and Linkage-disequilibrium Iteratively Nested Keyway (BLINK) model has shown greater statistical power by not requiring causal variants to be distributed across the genome, unlike the Settlement of MLM Under Progressively Exclusive Relationship (SUPER) model and the Fixed and Random Model Circulating Probability Unification (FarmCPU) model, and also reduces the number of false positives. The FarmCPU model, in particular, increases statistical power by reducing confounding between testing markers and kinship while controlling false positives^20^.

In this context, GWAS was performed using WGRS data from the mini-core collection, combined with phenotypic data generated for Fe, Zn and PC. Haplotype information is beneficial for GWAS to identify marker-phenotype associations^21^, to compensate bi-allelic limitation of SNP markers, and greatly enhance QTL detection efficiency^22,23^. Moreover, the haplotype-based analysis helps to control false positives and reveals the complex mechanism of causal haplotypes more effectively compared to individual SNPs^24^. Hence, this study was conducted to identify superior haplotypes, in addition to significant MTAs and candidate genes associated with Fe, Zn and PC homeostasis in groundnut kernels. These candidate genes, encoding various important regulatory proteins, can serve as targets for the development and validation of molecular markers. The discovery of superior haplotypes and donor genotypes marks the foundational steps to facilitate haplotype-based breeding (HBB) for enhancing kernel Fe, Zn, and PC in groundnut.

## Materials and methods

### Multi-environment phenotyping for Fe, Zn and PC

This study used 178 lines from the groundnut mini-core collection developed by ICRISAT Genebank^25^ (Supplementary Table 1). Experiments were conducted at ICRISAT, Patancheru, India (L1) across five seasons; rainy 2009 (S2), post-rainy 2009–10 (S3), post rainy 2010–11 (S4)^22^, and later during rainy 2020 (S5) and rainy 2021 (S6) for estimation of Fe and Zn (ppm) (Supplementary Table 2, 5). PC was estimated during four seasons at ICRISAT (L1)- rainy 2008 (S1), rainy 2009 (S2)^26^, rainy 2020 (S5) and rainy 2021 (S6). Additionally, PC data for two rainy seasons; 2008 (S1) and 2009 (S2) was also generated at Dharwad (L2)^27^. Frequency distribution graphs (violin plots) and Pearson correlation plot for Fe, Zn and PC were generated using “SRplot”^28^, while the principal component analysis (PCA)- biplot was produced using the “Factoextra”^29^ package in R software.

Fe and Zn contents were measured using the ICP-OES method^30^. For each genotype, 5–10 gm of seeds (15–20 seeds) were used. Approximately 0.3 g of oven-dried samples were placed in 50 ml labelled polypropylene tubes. Nitric acid (2 ml) and hydrogen peroxide (0.5 ml) were added, and the tubes allowed to withstand at the room temperature for overnight. Next day, tubes were vortexed and digested in a block for 30 min at 80 °C followed by 120 min for 125 °C. Caps were loosened after the first 30 min to equalize pressure. After cooling to room temperature, 25 ml distilled water was added and samples were mixed on an orbital shaker, filtered, and the supernatant analyzed using ICP-OES^5^. The phenotypic data for Fe and Zn in S2—S4 were analyzed at Charles Renard analytical laboratory, ICRISAT, while S5 and S6 samples were analyzed at NCML laboratory, Hyderabad.

The PC (%) was quantified using Near infrared-reflectance spectroscopy (NIRS). The groundnut samples (approximately 60 g) were scanned with a diode array analyzer at wavelengths from 950 to 1650 nm. Absorbance readings were recorded in 5 nm increments, and each sample was scanned three times to ensure accuracy^5^. Samples from two seasons S1 and S2, collected from L1 and L2, were analyzed using NIRS. The estimation of PC during the two seasons, S5 and S6 was done using NIRS at NCML laboratory, Hyderabad.

### Genomic DNA extraction, sequencing and SNP calling

The NucleoSpin® Plant II kit (Macherey–Nagel, Germany) was used to extract the genomic DNA from 100 mg of leaf sample tissue. 500 μL of lysis buffer was used to homogenize the leaf sample, and then 10 μL RNase was added to remove the RNA impurities. The samples were incubated at 65ºC for 1 h in water bath, then centrifuged at 5500 rpm for 10 min. A separate tube was used to collect the supernatant, and binding buffer of 450 μL was added. This whole mixture was centrifuged at 6000 rpm for 1 min after being filtered using a NucleoSpin plant MN column. In the final step, pre-heated elution buffer of 50 μL was added to the column’s membrane filter, incubated at 65ºC for 5 min, and centrifuged at 6000 rpm for 1 min to elute the DNA^31^. 0.8% agarose gel was used to check the DNA quality whereas the quantity of DNA was assessed using NanoDrop 8000 Spectrophotometer.

The TruSeq Library Prep Kit (Illumina, USA) was used to generate paired-end sequencing libraries with a 500 bp insert size to represent each genotype. Short reads (150 bp) with paired ends were generated at the Illumina HiSeq 2500 platform. Sequence data is provided under bio-project ID (PRJNA1002116, PRJNA490835 and PRJNA490832). After sequencing, adapter sequences were eliminated, and fastp v0.20.1 was used to filter low-quality reads^32^. Reads which had more than 5% undetermined nucleotides ("N") or greater than 20% low-quality bases (Phred quality score < 15) were not included. BWA-MEM v0.7.12-r1039^33^ was used to align the remaining high-quality reads with the reference genome of the cultivated *Arachis hypogaea* cv. Tifrunner (Tifrunner.gnm1.KYV3)^34^, using the parameters mem -M -R. SAMtools v1.21 was used to sort the alignments, and Picard v2.18.11 (http://picard.sourceforge) was used to detect duplicate reads. SOAPnuke v1.5.6 was used to filter the raw reads of each line. GATK v4.3.0.0 was used to perform SNP and InDel calling utilizing a joint calling approach^35^. Primarily HaplotypeCaller was used to generate genomic variant call format (GVCF) files for each sample, using the parameters: -T HaplotypeCaller -ERC GVCF –phred-scaled-global-read-mismapping-rate 45. CombineGVCFs were then used to carry out joint variant calling. Based on GATK best practices, VariantFiltration was used to refine the SNP set using the following hard filtering criteria: QD < 2.0, MQ < 40.0, FS > 60.0, ReadPosRankSum < − 8.0, MQRankSum < − 12.5, and SOR > 3.0. Using BamDeal v0.22 (https://github.com/BGI-shenzhen/BamDeal), depth was computed in 100-kb sliding windows in order to remove variants identified in regions with unusual sequencing depth. SnpEff v4.3t was used with default parameters to annotate the synonymous and non-synonymous SNPs. VCFtools v0.1.13 was used to filter out SNPs with a minor allele frequency below 0.05 or a missing rate larger than 0.2, using the parameters -max-missing 0.2 –maf 0.05.

### GWAS for Fe, Zn and PC

GWAS analysis was conducted using multi-season phenotyping data from 178 mini-core lines and 2,55,144 polymorphic SNPs from WGRS data. The Genome Association and Prediction Integrated Tool (GAPIT version 4.3.3)^36^ in “R” software package was employed to identify significant MTAs. In this study, several popular GWAS models were tested, including BLINK, MLMM, SUPER, the Compressed MLM (CMLM), FarmCPU, the General Linear Model (GLM) and MLM.

The significant MTAs were determined using Bonferroni correction, (p ≤ 0.05/N, where N = number of SNPs)^37^. Thresholds were set at 1.96 × 10⁻⁷ to remove false associations. Manhattan plots were generated, and MTAs above threshold were considered significant. Quantile–quantile (Q–Q) plots were used to identify the best-fit models for each trait and to confirm non-random associations of MTA loci with Fe, Zn, and PC.

### Identification of candidate genes from MTAs for Fe, Zn and PC

Candidate genes associated with the significant MTAs were identified using the GFF3 annotation file of *Arachis hypogaea* cv. Tifrunner (Tifrunner V1) obtained from peanutbase (https://www.peanutbase.org). Genomic regions associated with traits were examined, and genes with known roles in Fe, Zn, and PC homeostasis were prioritized.

The expression of the candidate genes was examined using the *Arachis hypogaea* gene expression atlas (AhGEA)^38^. Expression data were analysed for 16 tissues (Pre-soaked seeds, emerging radicle, cotyledons, embryo, root seedling, leaves at vegetative stage, root at vegetative stage, shoot at vegetative stage, shoot seedling, immature bud, seeds at 5 days after maturity (DAM), seeds at 15 DAM, seeds at 25 DAM, immature pod wall, mature pod wall, nodules). This analysis provides insights into functional relevance of candidate genes in homeostasis of Fe, Zn and PC.

### KASP marker development and validation

The SNPs associated with Fe were identified on 6 different chromosomes- (Ah03, Ah05, Ah14, Ah18, Ah19, Ah20), while one SNP associated with Zn was located on chromosome Ah05. These SNPs showed polymorphism between alleles associated with high vs. low Fe and Zn. SNPs were used to design KASP markers based on 300-bp flanking sequences^39^ (Supplementary Table 3). For each SNP marker, KASP assays were designed using two allele-specific forward primers and one common reverse primer. The developed KASP markers were validated in a diverse ICRISAT germplasm panel comprising 30 high-Fe/Zn lines and 20 low-Fe/Zn lines (Supplementary Table 4).

### Haplotype analysis, Haplo-pheno analysis and identification of superior haplotypes for Fe, Zn and PC

The subset of up to ten representative SNPs that are in linkage disequilibrium (LD) with the lead SNP discovered by the GWAS study, were used to conduct the haplotype analysis. These representative SNPs were selected to cover a range of LD levels with the significant SNP: low LD (r^2^ > 0.30 and ≤ 0.50), moderate LD (r^2^ > 0.50 and ≤ 0.70), and high LD (r^2^ > 0.70). This LD binning method captured the unique haplotype block patterns around the lead SNP by identifying the SNPs with various degrees of correlation. These representative SNPs are used to define the haplotype groups based on the unique genotype combinations observed within the panel ensuring that all distinct patterns were fully represented.

The identified haplotype groups were visualised using FlapJack software^40^. "Haplo-pheno" analysis was then carried out to evaluate the phenotypic effect of each haplotype. The phenotypic means of all the haplotype groups- which included at least three genotypes, were compared across multiple ranges. One-way analysis of variance (ANOVA), followed by the Tukey’s Honest Significant Difference (HSD) post-hoc test is performed to determine the significant differences among the groups. The haplotype or haplotype groups with the highest/preferred phenotypic mean values were considered as superior haplotypes and the haplotype groups with lowest mean values were considered as inferior haplotypes.

## Results

### Phenotypic variability, correlation and multi-variate association among Fe, Zn and PC

**Fig. 1:**
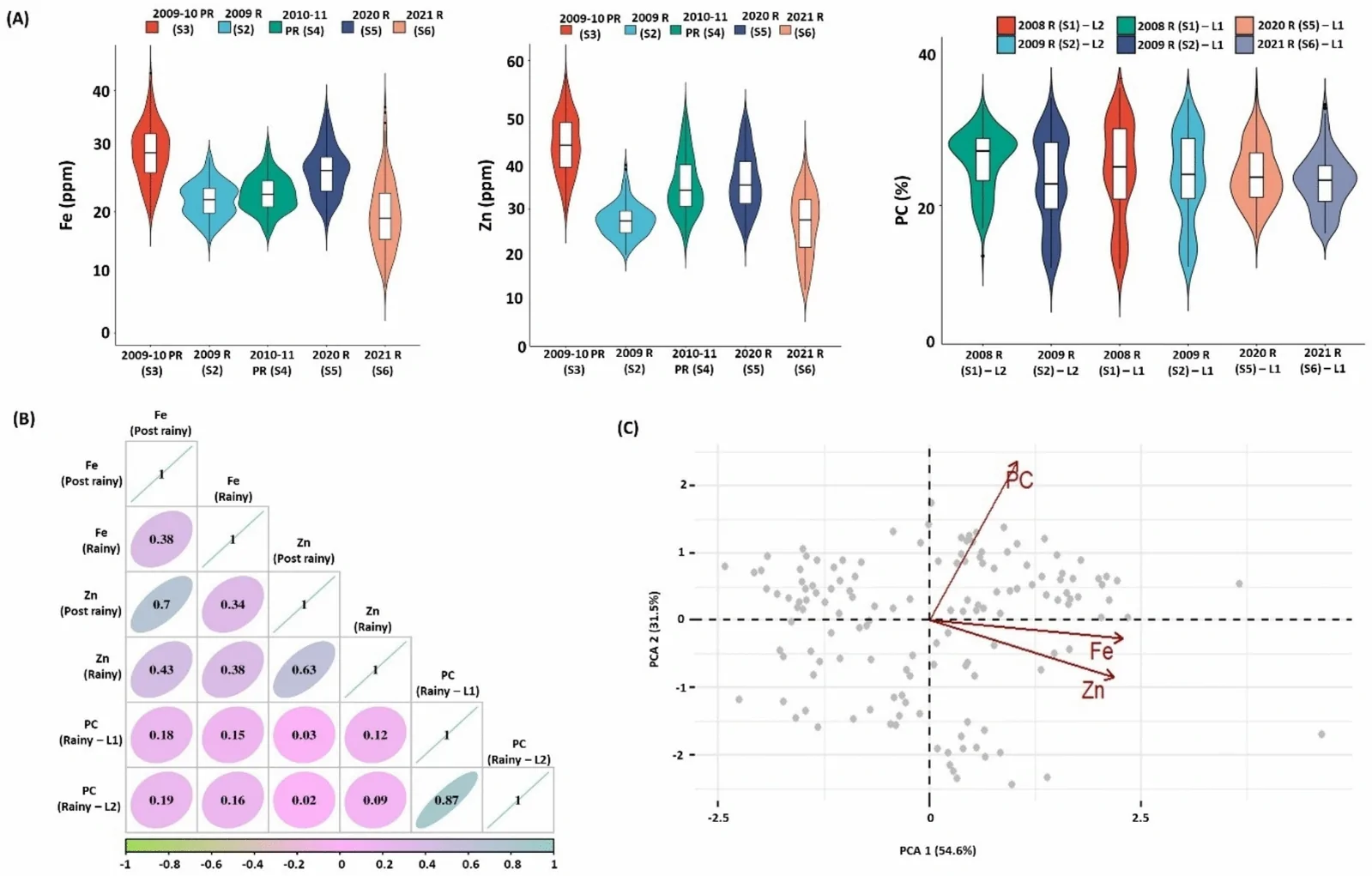
Phenotypic variation for Fe, Zn and PC in the diverse mini-core set. (**A**) Violin plots showing the distribution of Fe (ppm), Zn (ppm), and PC (%) for various genotypes across different years under both rainy (R) and post-rainy (PR) seasons. The box plot represents the average frequency of distribution of genotypes in a particular season (**B**) The correlation matrix indicating relationships among Fe, Zn and PC across seasons and locations, with significant positive correlation between Fe and Zn in pooled post-rainy season (*r* = 0.7) (**C**) Principal component analysis (PCA) demonstrating the distribution of the groundnut accessions (each dot on the plot represents an accession), based on the first two principal components, with PCA1 accounting for 54.6% of the variance and PCA2 accounting for 31.5%, cumulatively explaining 86.1% of the total variability.

The phenotypic distributions of Fe, Zn and PC across multiple seasons were visualized using violin plots (Fig. 1A), which showed wide variability. The mean values of Fe content varied across seasons as 21.9 ppm in S2, 29.8 ppm in S3, 23 ppm in S4, 26.7 ppm in S5 and 19.6 ppm in S6. Similarly, mean Zn values were 27.7 ppm in S2, 43 ppm in S3, 34.8 ppm in S4, 35.5 ppm in S5 and 27.2 ppm in S6. Likewise, the PC also exhibited considerable variation, with mean values of 25.1% in S1, 22.8% in S2 at L1 and 23.9% in S1, 23.3% in S2 at L2, 23.6% in S5 and 22.7% in S6 at L1. Across all seasons, Zn consistently exceeded Fe levels. Fe ranged from 7.56 ppm in S6 to 42.8 ppm in the S3, whereas Zn ranged from 10.9 ppm in S6 and to 62.35 ppm in S5. The PC values ranged from a minimum value of 12.74% in S1 at L2 to a maximum of 33.59% in S5 at L1.

Correlation analysis (Fig. 1B) showed a significant positive correlation among Fe, Zn and PC. The strongest correlation was observed between Fe (post rainy) and Zn (post rainy) (*r* = 0.7, *p* ≤ 0.05), followed by Fe (post rainy) and Zn (rainy) (*r* = 0.43, *p* ≤ 0.05). Partial but significant positive associations were also observed between Fe (rainy) and PC (rainy- ICRISAT) (*r* = 0.15, *p* ≤ 0.05), followed by Fe (rainy) and PC (rainy- Dharwad) (*r* = 0.16, *p* ≤ 0.05). Similar associations were observed between Zn (rainy) and PC (rainy- L1) (*r* = 0.12, *p* ≤ 0.05), and between Zn (rainy) and PC (rainy- L2) (*r* = 0.09, *p* ≤ 0.05).

PCA was performed using the three traits. The scatter plot (Fig. 1C) depicts the distribution of genotypes into two subgroups, revealed variations within these subgroups. The first principal component (PCA 1) (x-axis) accounted for 54.6% of total variance, while the second principal component (PCA 2) (y-axis) accounted for 31.5%, together explaining 86.1% of the total variation. The Fe and Zn across all seasons contributed positively to PCA 1, whereas PC contributed positively to both PCA 1 and PCA 2. The Fe and Zn contents were nearly parallel to each other, indicating a strong positive correlation, whereas, the PC was oriented differently from both the Fe and Zn, indicating a weaker positive correlation.

### Identification of MTAs

GWAS analysis with average data considering the mean of the accessions across different environments identified 15 significant MTAs for the three traits (4 MTAs for Fe, 4 for Zn, and 7 for PC) (Table 1). The highest number of MTAs were observed on chromosome Ah17 (3 MTAs), followed by 2 MTAs each on chromosomes Ah02, Ah13, and Ah19. Using seasons wise data, a total of 44 significant MTAs were detected- 20 MTAs for Fe, 13 for Zn and 11 for PC (Table 2). The maximum number of MTAs were identified on chromosome Ah03 (8 MTAs), followed by Ah17 (5 MTAs) and Ah05 and Ah19 chromosomes (4 MTAs each).

**Table 1:** List of MTAs detected for Fe, Zn and PC using different models for pooled season data.

Traits	Significant MTAs	Chromosome no	Position (bp)	Allele (Ref)	Allele (Alt)	*P-value*	SNP effect	PVE %	Models	Gene ID	Gene annotation
Iron	Arahy02_5010027	Ah02	5010027	G	C	1.23E−09	2.61	26.57	MLMM, SUPER, BLINK	*Arahy.LV3APC*	*NAC domain protein (NAC domain)*
										*Arahy.BEB4AA*	*P-loop containing nucleoside triphosphate hydrolases superfamily protein*
	Arahy13_17399102	Ah13	17399102	A	T	3.96E−08	2.28	11.27	BLINK	*Arahy.7P97F6*	*Zn finger, RING/FYVE/PHD-type, P-loop containing nucleoside triphosphate hydrolase*
										*Arahy.LJ25B0*	*Glycine-rich cell wall structural protein*
	Arahy19_27281401	Ah19	27281401	C	T	1.80E−09	2.38	18.18	MLMM, SUPER	*Arahy.QI0PHV*	*MYB transcription factor*
										*Arahy.QM0PMG*	*Zn finger MYM-type protein*
	Arahy20_80483965	Ah20	80483965	C	G	1.20E−08	− 3.76	14.04	BLINK	*Arahy.BF7CWQ*	*Uncharacterized protein*
										*Arahy.11RBMI*	*Protein serine/threonine kinase, Protein kinase-like domain*
Zinc	Arahy02_80149889	Ah02	80149889	G	A	1.38E−07	− 2.35	14.83	FarmCPU, GLM, MLM, CMLM	*Arahy.M50AVM*	*Autophagy-related protein*
										*Arahy.9R964H*	*Zn finger MYM-type protein*
	Arahy16_114034439	Ah16	114034439	A	G	1.19E−14	− 4.03	20.49	FarmCPU, GLM, MLM, CMLM	*Arahy.J1F3AY*	*P-loop containing nucleoside triphosphate hydrolase*
										*Arahy.1I6ZSS*	*MYB family transcription factor*
	Arahy17_102733285	Ah17	102733285	C	A	3.28E−09	2.73	0.71	FarmCPU, GLM, MLM, CMLM, BLINK	*Arahy.6K40U8*	*Dolichyl-diphosphooligosaccharide–protein glycosyltransferase subunit*
										*Arahy.XM7FE2*	*Maestro heat-like repeat-containing protein*
	Arahy18_127902116	Ah18	127902116	C	A	2.40E−10	2.49	12.48	FarmCPU, GLM, MLM, CMLM	*Arahy.V0ITCC*	*Unknown protein*
										*Arahy.I3B88T*	*RING finger* and* CHY Zn finger protein*
Protein	Arahy03_125540699	Ah03	125540699	T	G	4.70E−12	3.31	16.05	FarmCPU, GLM, MLM, CMLM, BLINK	*Arahy.VMLN7L*	*Protein serine/threonine kinases*
										*Arahy.B3BEZ6*	*protein serine/threonine kinases*
	Arahy11_48679516	Ah11	48679516	G	C	1.99E−08	2.32	5.42	FarmCPU, GLM, MLM, CMLM	*Arahy.Y46FKN*	*Unknown protein*
	Arahy12_56160933	Ah12	56160933	T	G	4.94E−09	− 1.31	4.03	BLINK	*Arahy.V0ZW9L*	*NEP1-interacting protein*
										*Arahy.B5VTGG*	*Nucleotide-binding, nucleic acid binding*
	Arahy13_58948078	Ah13	58948078	T	G	2.67E−08	− 1.62	1.84	FarmCPU, GLM, MLM, CMLM	*Arahy.5L4UK7*	*Nudix hydrolase*
										*Arahy.0IU8HI*	*Protein kinase family protein*
	Arahy17_10878383	Ah17	10878383	A	G	5.44E−10	0.94	7.03	BLINK	*Arahy.KX8RC4*	*2-oxoglutarate (2OG)* and* Fe (II)-dependent oxygenase superfamily protein*
										*Arahy.4D7KBI*	*Protein kinase superfamily protein*
	Arahy17_110193095	Ah17	110193095	G	A	3.83E−08	− 2.58	6.74	CMLM, FarmCPU, GLM, MLM	*Arahy.PE3CF6*	*E3 ubiquitin-protein ligase*
	Arahy19_28004976	Ah19	28004976	T	C	5.72E−10	3.10	4.17	CMLM, FarmCPU, GLM, MLM	*Arahy.QVG73I*	*Phosphomevalonate kinase*
										*Arahy.X0A8D9*	*Aspartic proteinase; Aspartic peptidase, Saposin-like, Aspartic peptidase domain*

**Table 2:** List of MTAs detected for Fe, Zn and PC using different models for season-wise data.

Season	Trait	Significant MTAs	Type of variant	Chromosome No	Position (bp)	Allele (Ref)	Allele (Alt)	P Value	SNP effect	PVE %	Models	Gene ID	Gene annotations
S3	Iron	Ah19_121223186	Intergenic region	Ah19	121223186	T	C	9.82E−08	1.91	2.51	SUPER, MLMM	*Arahy.H66LJ0*	*Polyprotein Citrus endogenous pararetrovirus*
												*Arahy.H3HGUT*	*MYB transcription factor*
S3	Iron	Ah20_80483965	Intergenic region	Ah20	80483965	C	G	1.53E−10	− 2.69	53.43	BLINK, FarmCPU	*Arahy.BF7CWQ*	*Uncharacterized protein*
												*Arahy.11RBMI*	*ATP-binding/protein serine/threonine kinase*
S2	Iron	Ah03_28583444	Downstream gene variant	Ah03	28583444	C	T	1.47E−13	1.44	15.37	BLINK, SUPER, MLMM	*Arahy.9BBP6D*	*L-lactate/malate dehydrogenase*
S2	Iron	Ah03_105594763	Intergenic region	Ah03	105594763	C	T	4.84E−12	1.74	1.24	FarmCPU, BLINK, MLMM	*Arahy.H3WGQZ*	*Unknown protein*
												*Arahy.MYB15F*	*Protein LURP-one-related 17-like*
S2	Iron	Ah04_18847124	Intergenic region	Ah04	18847124	C	T	3.77E−12	2.06	9.68	BLINK	*Arahy.CT4NDI*	*Heavy metal transport/detoxification superfamily protein*
												*Arahy.I3MG1X*	*Histone-lysine N-methyltransferase ATXR2-like isoform*
S2	Iron	Ah05_10145205	Intergenic region	Ah05	10145205	G	A	8.47E−08	− 1.53	1.12	SUPER	*Arahy.XN4TZ5*	*Plasma membrane mannitol transporter*
												*Arahy.GVE3NJ*	*Unknown protein*
S2	Iron	Ah10_32538050	Intergenic region	Ah10	32538050	T	A	2.78E−08	1.40	2.33	FarmCPU	*Arahy.ZME7HS*	*Uncharacterized protein*
												*Arahy.DGD0VB*	*Oxysterol-binding protein-related protein*
S2	Iron	Ah11_72760600	Intergenic region	Ah11	72760600	G	A	5.53E−10	1.69	9.36	BLINK, MLMM	*Arahy.W1WWEB*	*Homolog of nucleolar protein (Nop domain)*
												*Arahy.XPYE9K*	*ATP phosphoribosyl transferase*
S2	Iron	Ah17_7608976	Intergenic region	Ah17	7608976	G	A	2.91E−08	1.16	2.55	FarmCPU	*Arahy.4B5N5U*	*Receptor-like protein kinase; protein serine/threonine kinase*
												*Arahy.6Q0Z4R*	*Zinc finger MYM-type protein*
S2	Iron	Ah17_131484790	Upstream gene variant	Ah17	131484790	C	T	1.35E−08	1.50	1.99	FarmCPU	*Arahy.Q0I0E2*	*NADPH-cytochrome family reductase*
S2	Iron	Ah18_122103781	Intergenic region	Ah18	122103781	C	T	1.10E−07	2.11	14.63	SUPER	*Arahy.Q4GIHG*	*Zinc finger MYM-type protein*
												*Arahy.USIX8K*	*5-methylthioadenosine/S-adenosylhomocysteine deaminase*
S2	Iron	Ah20_70265096	Intergenic region	Ah20	70265096	G	C	9.49E−09	− 0.46	22.38	BLINK	*Arahy.ZBIL8C*	*Cyclophilin-like peptidyl-prolyl cis–trans isomerase family protein*
												*Arahy.UCCR5R*	*Actin-binding FH2 (Formin Homology) protein*
S4	Iron	Ah09_107364323	Upstream gene variant	Ah09	107364323	C	T	5.29E−08	1.09	5.44	SUPER	*Arahy.CUT3DD*	*Transcriptional activator DEMETER-like, DNA glycosylase*
S4	Iron	Ah13_58751943	Intergenic region	Ah13	58751943	A	T	8.47E−08	1.38	7.93	SUPER	*Arahy.B2ND5Q*	*Unknown protein*
												*Arahy.5L4UK7*	*Nudix hydrolase homolog 14*
S4	Iron	Ah17_12433761	Intergenic region	Ah17	12433761	C	T	3.44E−08	1.44	12.23	SUPER	*Arahy.13YYB8*	*Unknown protein*
												*Arahy.3DA9LK*	*Transport inhibitor response like protein*
S4	Iron	Ah18_55925435	Intergenic region	Ah18	55925435	T	C	2.54E−09	2.36	18.75	MLMM, SUPER	*Arahy.6ZMA90*	*Novel plant snare 12*
												*Arahy.MQHC27*	*Actin-binding FH2 (Formin Homology) protein*
S4	Iron	Ah19_135254456	Intergenic region	Ah19	135254456	C	T	9.11E−08	1.04	1.03	SUPER	*Arahy.2U0JEX*	*UDP-Glycosyltransferase superfamily protein*
												*Arahy.999YA8*	*Calcium-dependent lipid-binding family protein*
S4	Iron	Ah19_145627456	Intergenic region	Ah19	145627456	T	C	1.74E−08	− 1.00	5.35	SUPER	*Arahy.72B7PK*	*L-type lectin-domain containing receptor kinase*
												*Arahy.I6PPQF*	*Probable protein phosphatase*
S5	Iron	Ah14_5692509	Upstream gene variant	Ah14	5692509	A	G	1.41E−07	14.85	65.38	FarmCPU, BLINK	*Arahy.NX1XST*	*Dihydroxy-acid dehydratase*
S6	Iron	Ah03_87593234	Intergenic region	Ah03	87593234	T	G	9.09E−10	5.51	51.21	BLINK, MLMM, FarmCPU	*Arahy.HNJF4G*	*26S proteasome regulatory subunit like isoform*
												*Arahy.9RX38M*	*DCD (Development* and* Cell Death) domain protein*
S3	Zinc	Ah05_35417862	Intergenic region	Ah05	35417862	T	C	5.09E−08	− 1.89	55.01	MLMM	*Arahy.AN6HNQ*	*Uncharacterized protein*
												*Arahy.GRNJ5W*	*Zinc finger, RING/FYVE/PHD-type*
S3	Zinc	Ah05_27023896	Intergenic region	Ah05	27023896	T	C	1.04E−10	− 1.11	67.42	BLINK	*Arahy.F1RJAK*	*Formin, FH2 domain*
												*Arahy.G26LFA*	*Phosphorelay response regulator*
S2	Zinc	Ah03_28583444	Downstream gene variant	Ah03	28583444	C	T	5.29E−13	1.41	13.25	BLINK, FarmCPU	*Arahy.9BBP6D*	*L-lactate/malate dehydrogenase*
S2	Zinc	Ah05_15332507	Intergenic region	Ah05	15332507	C	T	7.09E−09	1.75	12.08	BLINK	*Arahy.TLT0RJ*	*Zinc finger, CCHC-type*
												*Arahy.29A50M*	*Myc-type, basic helix-loop-helix (bHLH) domain*
S2	Zinc	Ah06_100584371	Intergenic region	Ah06	100584371	C	T	7.27E-−11	− 1.50	10.72	BLINK	*Arahy.NWPU6Y*	*Uncharacterized protein*
												*Arahy.12HUMH*	*NAC domain protein*
S2	Zinc	Ah07_51562128	Upstream gene variant	Ah07	51562128	A	G	1.17E−09	3.23	16.89	MLMM	*Arahy.6YW6LE*	*Aldo/keto reductase family oxidoreductase; NADP-dependent oxidoreductase domain*
S2	Zinc	Ah09_7062575	Intergenic region	Ah09	7062575	C	A	1.74E−10	− 1.90	1.65	FarmCPU	*Arahy.C4WT98*	*P-loop containing nucleoside triphosphate hydrolase*
												*Arahy.KAVD32*	*Ethylene insensitive 3 family protein*
S2	Zinc	Ah10_86959972	Intron variant	Ah10	86959972	G	A	2.35E−10	− 1.97	29.06	BLINK	*Arahy.4HXN36*	*Myb/SANT-like DNA-binding domain protein*
S2	Zinc	Ah15_103380195	Intergenic region	Ah15	103380195	A	G	2.23E−09	2.03	8.40	BLINK	*Arahy.QXL28E*	*GDSL-like Lipase/Acylhydrolase superfamily protein*
												*Arahy.YMBN2Y*	*GDSL-like Lipase/Acylhydrolase superfamily protein*
S2	Zinc	Ah19_84532565	Intergenic region	Ah19	84532565	G	A	5.55E−09	1.31	6.61	BLINK	*Arahy.N0IL4Q*	*Phosphoribosylaminoimidazole carboxylase*
												*Arahy.6PZ0D3*	*DNA mismatch repair protein, UbiB domain*
S4	Zinc	Ah13_120211775	Intergenic region	Ah13	120211775	G	A	1.69E−09	− 3.52	38.05	BLINK	*Arahy.292F09*	*CLPTM1-like membrane protein*
												*Arahy.H71TAS*	*Calcium-dependent protein kinase*
S5	Zinc	Ah02_51505705	Intergenic region	Ah02	51505705	G	A	4.82E−30	− 29.22	99.12	BLINK, MLMM, CMLM, FarmCPU	*Arahy.U6R2AI*	*Uncharacterized protein*
												*Arahy.J9ASUV*	*Micronuclear linker histone polyprotein*
S5	Zinc	Ah02_46495272	Intergenic region	Ah02	46495272	C	A	9.91E−12	16.21	2.66	MLMM	*Arahy.7X6UW5*	*Uncharacterized protein*
												*Arahy.J1W7N5*	*Unknown protein*
S1 L2	Protein	Ah03_125540699	Intergenic region	Ah03	125540699	T	G	5.43E−11	3.28	25.69	FarmCPU, GLM	*Arahy.VMLN7L*	*Protein serine/threonine kinases*
												*Arahy.B3BEZ6*	*Protein serine/threonine kinases*
S1 L2	Protein	Ah13_18601962	Intergenic region	Ah13	18601962	G	T	2.93E−08	1.88	5.54	FarmCPU	*Arahy.NG0HJG*	*Unknown protein*
												*Arahy.H2FU9Z*	*Unknown protein*
S1 L2	Protein	Ah17_49411056	Intergenic region	Ah17	49411056	A	G	2.46E−08	− 2.34	1.07	FarmCPU	*Arahy.P38DRG*	*Unknown protein*
												*Arahy.DD6N9K*	*Protein tyrosine/serine/threonine phosphatase*
S1 L2	Protein	Ah17_133010553	Intron variant	Ah17	133010553	C	T	3.47E−09	− 3.07	8.53	FarmCPU	*Arahy.RFW94S*	*Syntaxin, putative;*
S2 L2	Protein	Ah03_125540699	Intergenic region	Ah03	125540699	T	G	8.41E−12	4.40	10.95	GLM, FarmCPU	*Arahy.VMLN7L*	*Protein serine/threonine kinases*
												*Arahy.B3BEZ6*	*Protein serine/threonine kinases*
S2 L2	Protein	Ah03_107178229	Intergenic region	Ah03	107178229	C	T	5.43E−10	− 3.97	0.50	MLMM	*Arahy.D5URA7*	*Novel plant SNARE*
												*Arahy.9A4AY7*	*MYB transcription factor*
S2 L2	Protein	Ah12_116861708	Upstream gene variant	Ah12	116861708	G	A	3.90E−08	4.19	18.70	MLMM	*Arahy.SITW69*	*FAD-binding Berberine family protein, FAD-binding, UDP-N-acetylmuramate dehydrogenase activity, flavin adenine dinucleotide binding*
S2 L2	Protein	Ah18_126543814	Upstream gene variant	Ah18	126543814	A	T	1.49E–−09	1.64	2.60	FarmCPU	*Arahy.19AV4I*	*P-loop containing nucleoside triphosphate hydrolase*
S1 L1	Protein	Ah03_125540699	Intergenic region	Ah03	125540699	T	G	1.20E−07	2.45	7.82	GLM	*Arahy.VMLN7L*	*Protein serine/threonine kinases*
												*Arahy.B3BEZ6*	*Protein serine/threonine kinases*
S1 L1	Protein	Ah03_68769471	Upstream gene variant	Ah03	68769471	G	A	1.59E−15	2.42	3.50	FarmCPU, GLM, MLMM	*Arahy.GBY2EB*	*Putative pectinesterase/pectinesterase inhibitor*
S1 L1	Protein	Ah15_49814667	Intergenic region	Ah15	49814667	G	T	6.05E−10	− 1.75	6.03	FarmCPU	*Arahy.0RJM1Q*	*UDP-glucose 6-dehydrogenase family protein*
												*Arahy.I0PDCZ*	*Cinnamyl alcohol dehydrogenase*
S1 L1	Protein	Ah19_6490479	Intergenic region	Ah19	6490479	C	T	9.35E−15	0.03	17.59	FarmCPU	*Arahy.7NK30A*	*Heat shock protein*
												*Arahy.KJ6PSZ*	*Heat shock protein*
S2 L1	Protein	Ah03_27122996	Upstream gene variant	Ah03	27122996	T	C	4.17E−09	− 2.14	5.24	FarmCPU	*Arahy.ZJ4Z2J*	*Protein serine/threonine kinase*
S2 L1	Protein	Ah03_125540699	Intergenic region	Ah03	125540699	T	G	7.81E−12	4.44	4.23	FarmCPU, GLM	*Arahy.VMLN7L*	*Protein serine/threonine kinases*
												*Arahy.B3BEZ6*	*Protein serine/threonine kinases*
S2 L1	Protein	Ah03_107178229	Intergenic region	Ah03	107178229	C	T	1.80E−11	− 3.97	54.30	MLMM	*Arahy.D5URA7*	*Novel plant SNARE*
												*Arahy.9A4AY7*	*MYB transcription factor*
S2 L1	Protein	Ah12_116861708	Upstream gene variant	Ah12	116861708	G	A	3.63E−09	4.11	20.01	MLMM	*Arahy.SITW69*	*FAD-binding Berberine family protein, FAD-binding, UDP-N-acetylmuramate dehydrogenase activity, flavin adenine dinucleotide binding*

Seasons: Rainy seasons 2008 (S1); Rainy seasons 2009 (S2); post-rainy 2009–10 (S3), post rainy 2010–11 (S4), rainy 2020 (S5) and rainy 2021 (S6), Locations: ICRISAT, Patancheru, (L1); Dharwad (L2).

#### Genomic associations identified for Fe

**Fig. 2:**
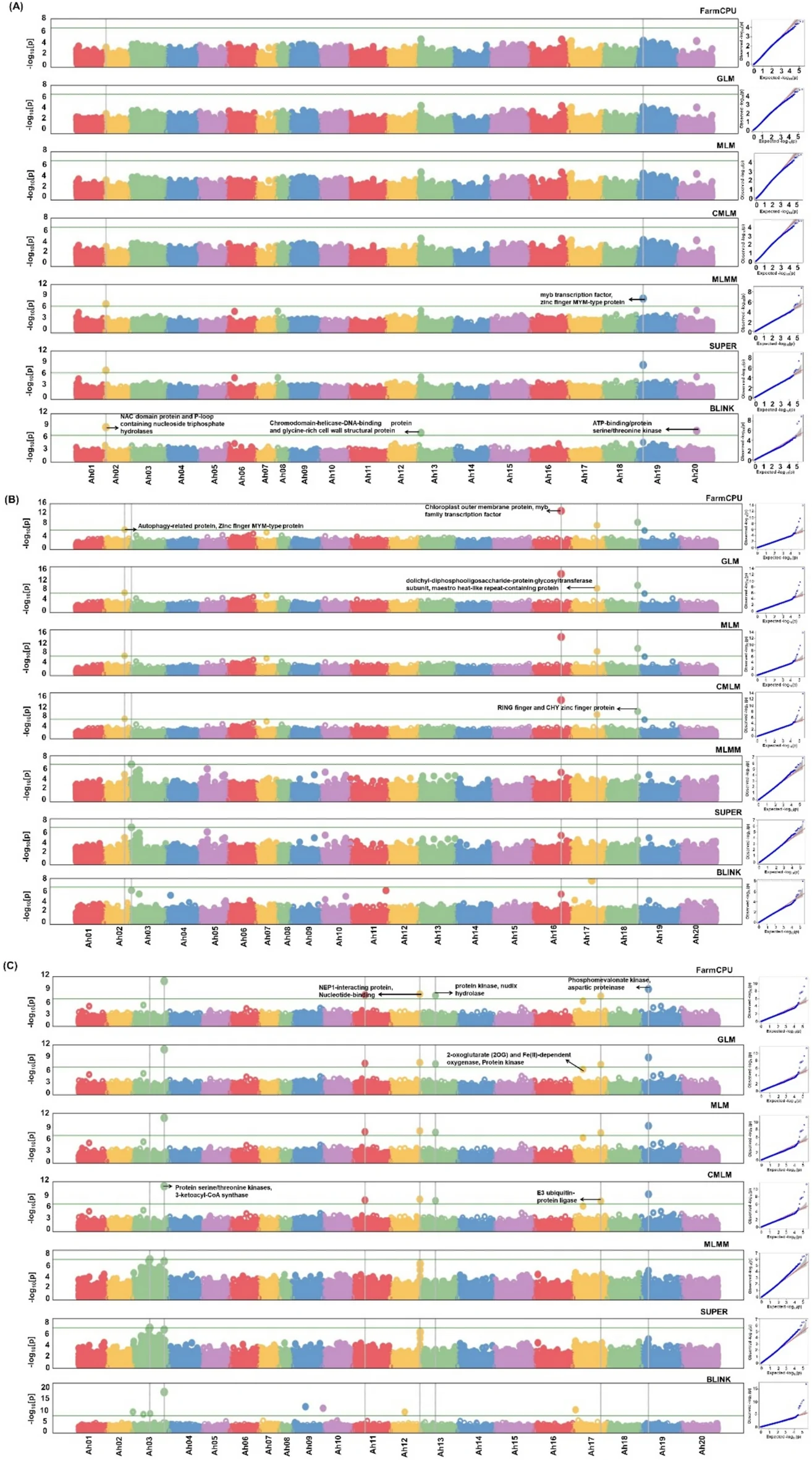
Manhattan plots and optimal Q-Q plots for (**A**) Fe (**B**) Zn and (**C**) PC for pooled season data. The horizontal dashed lines represent the significant threshold (*p* = 1 × 10^−7^, Bonferroni correction) and the vertical dashed lines represent the candidate genes at the significant MTAs associated with (**A**) Fe on chromosomes- Ah02, Ah13, Ah19 and Ah20 (**B**) Zn on chromosomes- Ah02, Ah16, Ah17 and Ah18 (**C**) PC on chromosomes- Ah03, Ah11, Ah12, Ah13, Ah17 and Ah19.

For pooled season data, 4 significant MTAs were identified for Fe on chromosomes Ah02 (Ah02_5010027), Ah13 (Ah13_17399102), Ah19 (Ah19_27281401) and Ah20 (Ah20_80483965) (Fig. 2A). Their *p-values* ranged from 1.20 × 10^–8^ (Ah20_80483965) to 1.80 × 10^–9^ (Ah19_27281401), explaining 14.04% to 18.18% of phenotypic variation. The SNPs Ah13_17399102 and Ah20_80483965 were uniquely identified by BLINK model. Notably, MTA (Ah02_5010027) associated with Fe, identified on chromosome Ah02 was detected by the models (BLINK, MLMM and SUPER), with the highest phenotypic variance of 26.57% and ‘*p*’ value of 1.23 × 10^–9^, whereas Ah13_17399102 was found to have the lowest phenotypic variance of 11.27% with a ‘*p*’ value of 3.96 × 10^–8^.

For seasonal analysis, 20 significant MTAs were identified for Fe on different chromosomes Ah03, Ah04, Ah05, Ah09, Ah10, Ah11, Ah13, Ah17, Ah18, Ah19 and Ah20. The *p-values* ranged from 1.10 × 10^–7^ (Ah18_122103781) to 1.47 × 10^–13^ (Ah03_28583444), explaining 14.63% to 15.37% of phenotypic variation. Three MTAs on chromosome Ah03 (Ah03_28583444, Ah03_105594763 and Ah03_87593234) were consistently identified by at least three models during 2009 rainy and 2021 rainy (Supplementary Fig. 1 and 2). The highest phenotypic variance (65.38%) was observed for Ah14_5692509 with a ‘*p*’ value of 1.41 × 10^–7^ followed by Ah20_80483965 (53.43%) with a ‘*p*’ value of 1.53 × 10^–10^.

#### Genomic associations identified for Zn

For pooled season data, 4 significant MTAs were identified for Zn on chromosomes Ah02 (Ah02_80149889), Ah16 (Ah16_114034439), Ah17 (Ah17_102733285), and Ah18 (Ah18_127902116) (Fig. 2B). All the MTAs associated with the Zn were detected by the models- FarmCPU, GLM, MLM and CMLM. The MTA Ah16_114034439 was observed with highest phenotypic variance of 20.49% and a *p*-value of 1.19 × 10^–14^, whereas Ah17_102733285 was observed with lowest phenotypic variance of 0.71% and a *p*-value of 3.28 × 10^–9^. The other MTAs, Ah02_80149889 and Ah18_127902116, located on chromosomes Ah02 and Ah18 were identified with phenotypic variance values of 14.83% and 12.48%, and ‘*p*’ values of 1.38 × 10^–7^ and 2.40 × 10^–10^, respectively.

Seasonal analysis identified 13 significant MTAs across 10 chromosomes. The *p-values* for these significant MTAs ranged from 5.09 × 10^–8^ (Ah05_35417862) to 4.82 × 10^–30^ (Ah02_51505705), explaining 55.01% to 99.12% of phenotypic variance. The MTA Ah02_51505705 on chromosome Ah02 was detected by the four models (BLINK, CMLM, FarmCPU, MLMM) during 2020 rainy (Supplementary Fig. 3 and 4). The highest phenotypic variance (99.12%) was observed for Ah02_51505705 with a ‘*p*’ value of 4.82 × 10^–30^ followed by Ah05_27023896 (67.42%) with a ‘*p*’ value of 1.04 × 10^–10^.

#### Genomic associations identified for PC

For PC, seven significant MTAs were identified for pooled season data across six chromosomes like Ah03 (Ah03_2532408), Ah11 (Ah11_48679516), Ah12 (Ah12_56160933), Ah13 (Ah13_58948078), Ah17 (Ah17_10878383 and Ah17_110193095), Ah19 (Ah19_28004976) (Fig. 2C). All the MTAs associated with PC were detected by FarmCPU, GLM, MLM and CMLM models. The *p*-values for these MTAs ranged from 1.99 × 10^–8^ (Ah11_48679516) to 5.72 × 10^–10^ (Ah19_28004976), explaining 5.42% to 4.17% of phenotypic variance. Interestingly, two MTAs coding for the PC were found on the chromosome Ah17 at two different positions. The highest phenotypic variance of 16.05% was observed for Ah03_125540699 with a ‘*p*’ value of 4.70 × 10^–12^ and the lowest phenotypic variance of 1.84% was observed for Ah13_58948078 with a ‘*p*’ value of 2.67 × 10^–8^.

For season-wise analysis, 11 significant MTAs were identified on seven chromosomes (Ah03, Ah12, Ah13, Ah15, Ah17, Ah18 and Ah19). The *p-values* for these significant MTAs ranged from 1.20 × 10^–7^ (Ah03_125540699) to 9.35 × 10^–15^ (Ah19_6490479), explaining 7.82% to 17.59% of phenotypic variance. The MTA Ah03_68769471 on chromosome Ah03 was consistently detected by the three models (FarmCPU, GLM, MLMM) (Supplementary Fig. 5 and 6).

### Identification of candidate genes and their expression analysis

A total of 28 candidate genes were identified for pooled season data, including 8 each for Fe and Zn while 12 for PC. The seasonal data analysis identified 62 genes, including 30 for Fe, 18 for Zn and 14 for PC, associated with the MTAs. These candidate genes were involved in different molecular, cellular, and biological functions related to the traits. These candidate genes encode various important proteins like *MYB transcription factor*, *Zn finger MYM type protein*, *RING finger MYM type protein*, *ATP binding/ threonine kinase/ protein serine/ protein kinase*, *NAC domain protein*, and *Glycine rich cell wall structural protein*. Several other genes like *Heavy metal transport/detoxification superfamily protein*, *ATP phosphoribosyl transferase*, *NADPH-cytochrome family reductase*, *Nudix hydrolase*, *Probable protein phosphatase*, *Calcium-dependent protein kinase*, *Heat shock protein*, *E3 ubiquitin-protein ligase*, *2-oxoglutarate* (*2OG*) and *Fe* (*II*)*-dependent oxygenase superfamily protein*, *Phosphomevalonate kinase*, *aspartic proteinase*, *3-ketoacyl-CoA synthase*, *Dolichyl-diphosphooligosaccharide-protein* and *Jasmonic acid carboxyl methyltransferase* were identified as candidate genes related to the trait functions and biological processes. The domains for the proteins encoded by these genes were either directly or indirectly related to the Fe, Zn and PC. The SNPs, Ah02_5010027 associated with *NAC domain protein*, Ah02_80149889, Ah18_127902116, Ah13_17399102, Ah17_7608976, Ah18_122103781, Ah05_35417862, Ah05_15332507 associated with *RING* and *Zn finger protein*, Ah16_114034439, Ah18_27281401, Ah19_121223186, Ah10_86959972 associated with *MYB transcription* were found linked to Fe and Zn homeostasis pathway.

**Fig. 3:**
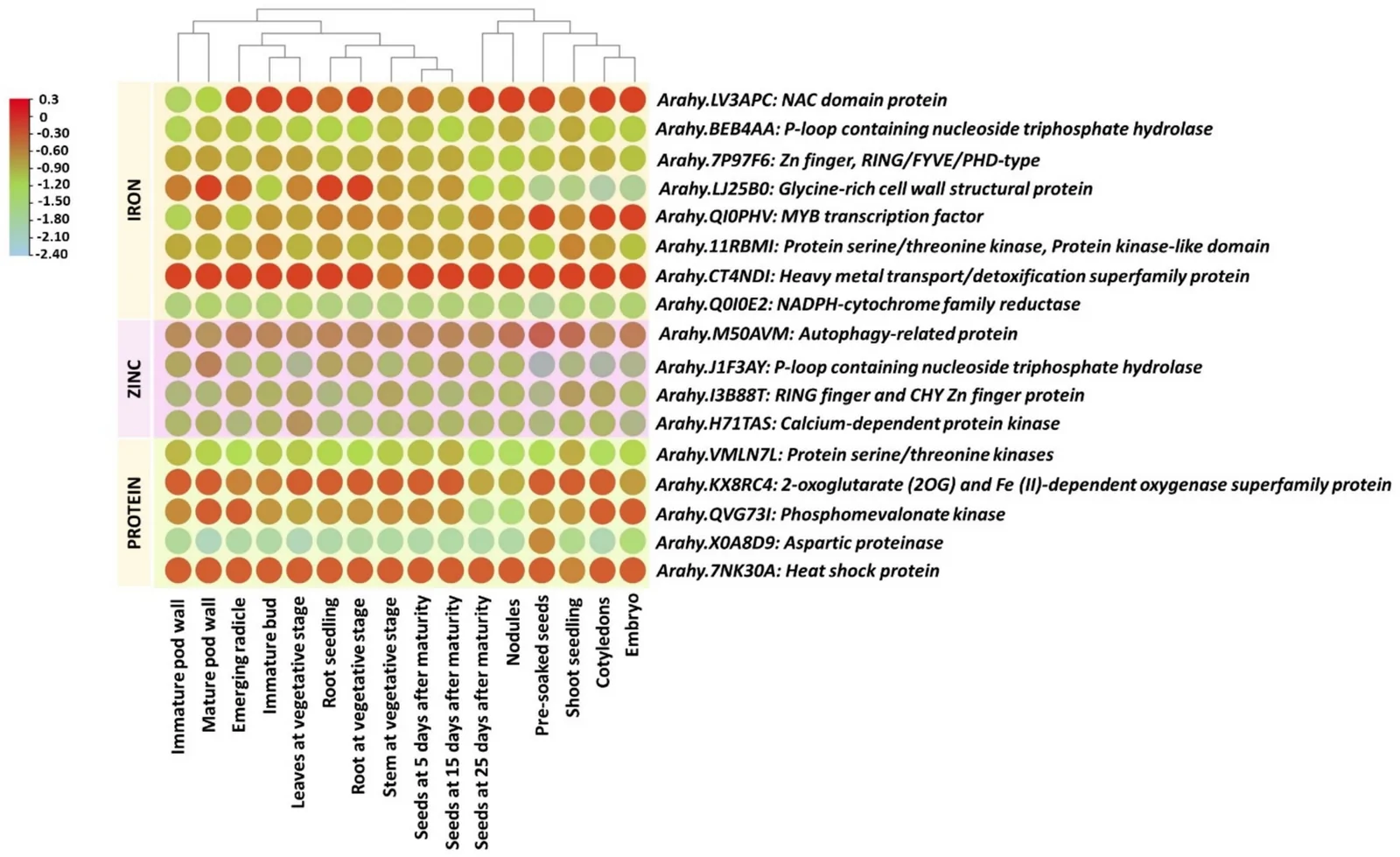
Tissue-specific expression study of candidate genes during different developmental stages based on AhGEA data. The expression of 17 candidate genes in 16 different tissues (Pre-soaked seeds, emerging radicle, cotyledons, embryo, root seedling, leaves at vegetative stage, root at vegetative stage, shoot at vegetative stage, shoot seedling, immature bud, seeds at 5 DAM, seeds at 15 DAM, seeds at 25 DAM, immature pod wall, mature pod wall, nodules) are plotted in the heatmap.

Among the total candidate genes identified, 17 genes showed expression during different developmental stages based on the *Arachis hypogaea* gene expression atlas (AhGEA)^38^ (Fig. 3). Gene expression studies using AhGEA show that *NAC domain protein* (*Arahy.LV3APC*), *Heavy metal transport/detoxification superfamily protein* (*Arahy.CT4NDI*), *2-oxoglutarate* (*2 OG*) and *Fe* (*II*)-*dependent oxygenase* (*Arahy.KX8RC4*) and *Heat shock protein* (*Arahy.7NK30A*) were found to show higher expression in majority of the plant tissues studied, whereas *Glycine rich cell wall structural protein* (*Arahy.LJ25B0*), *MYB transcription factor* (*Arahy.QI0PHV*) and *Phosphomevalonate kinase* (*Arahy.QVG73I*) showed higher expression in some specific plant tissues only.

### Allele mining for significantly associated SNPs

In the current study, significant MTAs for all three traits and their associated candidate genes were identified on different chromosomes. To utilize these MTAs and genes, SNP polymorphism was examined in a diverse mini-core collection.

Based on MTA positions associated with the three traits, allele mining was conducted to identify the alleles associated with high and low trait values in the diverse mini-core lines (Supplementary Fig. 7). The diverse set included 16 genotypes for Fe, 22 genotypes for Zn and 18 genotypes for PC. Among the 16 Fe genotypes, high Fe genotypes ranged from 28.7 to 52.5 ppm and low Fe genotypes ranged from 15.5 to 19.4 ppm. Among the 22 Zn genotypes, high Zn genotypes ranged from 40.1 to 49.2 ppm, while low Zn genotypes ranged from 19.8 to 27.9 ppm. Similarly, for 18 PC genotypes, high PC genotypes ranged between 26.8 to 28.6%, while low PC genotypes ranged from 18.7 to 19.8%.

Of the four MTAs associated with Fe, three MTAs -Ah02_5010027, Ah13_17399102 and Ah20_80483965 showed clear polymorphism between high and low Fe genotypes. Among the four MTAs identified for Zn, two MTAs -Ah16_114034439 and Ah18_127902116 showed clear polymorphism between the high and low Zn content lines. Similarly, among the seven MTAs identified for PC, two MTAs Ah03_125540699 and Ah19_28004976 showed clear polymorphism between high and low PC lines. Interestingly, genotype ICG 9249 with mean values of Fe-30.1 ppm, Zn-40.3 ppm and PC-27.3% carried alleles associated with high Fe, Zn and PC. In contrast, four genotypes, ICG 2777 (Fe- 19.4 ppm, Zn- 27.9 ppm), ICG 6813 (Fe- 19 ppm, Zn- 26.3 ppm), ICG 5016 (Fe- 18.4 ppm, Zn- 25.7 ppm) and ICG 14834 (Fe- 15.5 ppm, Zn- 19.8 ppm) carried alternate alleles for low Fe and Zn.

Likewise, in-silico validation of the MTAs identified from the season-wise analysis was done using diverse set of 40 genotypes for both Fe and Zn (Supplementary Fig. 8). Among the 20 MTAs associated with Fe, 8 MTAs- Ah05_10145205, Ah18_122103781, Ah20_70265096, Ah18_55925435, Ah19_135254456, Ah19_145627456, Ah14_5692509, Ah03_87593234 showed clear polymorphism between high and low Fe genotypes. Similarly, among the 13 MTAs associated with Zn, three MTAs- Ah05_35417862, Ah05_27023896 and Ah02_46495272 showed clear polymorphism between high and low Zn lines. Seven genotypes—ICG 14118 (Fe = 42.9 ppm, Zn = 53.3 ppm), ICG 15309 (Fe = 37.6 ppm, Zn = 52.9 ppm), ICG 36 (Fe = 36.4 ppm, Zn = 56.6 ppm), ICG 4911 (Fe = 35.2 ppm, Zn = 53.9 ppm), ICG 7963 (Fe = 34.7 ppm, Zn = 47.7 ppm), ICG 10092 (Fe = 34 ppm, Zn = 48.4 ppm), and ICG 1274 (Fe = 31.1 ppm, Zn = 47.7 ppm)—carried alleles associated with high Fe and Zn. In contrast, genotypes such as ICG 8760 (Fe = 20.9 ppm, Zn = 25.7 ppm), ICG 4412 (Fe = 20.6 ppm, Zn = 13.7 ppm), ICG 4156 (Fe = 20.3 ppm, Zn = 15.9 ppm), ICG 4389 (Fe = 19.7 ppm, Zn = 16.4 ppm), ICG 76 (Fe = 18.9 ppm, Zn = 14.2 ppm), ICG 4527 (Fe = 17.7 ppm, Zn = 15.3 ppm), ICG 13723 (Fe = 10.9 ppm, Zn = 14.8 ppm), and ICG 5016 (Fe = 9.2 ppm, Zn = 24.4 ppm) carried alleles associated with low Fe and Zn. These findings suggest that these identified SNPs can be effectively deployed for allele-specific marker development for marker-assisted selection (MAS) to improve nutritional quality in groundnut.

### Development and validation of KASP markers

**Fig. 4:**
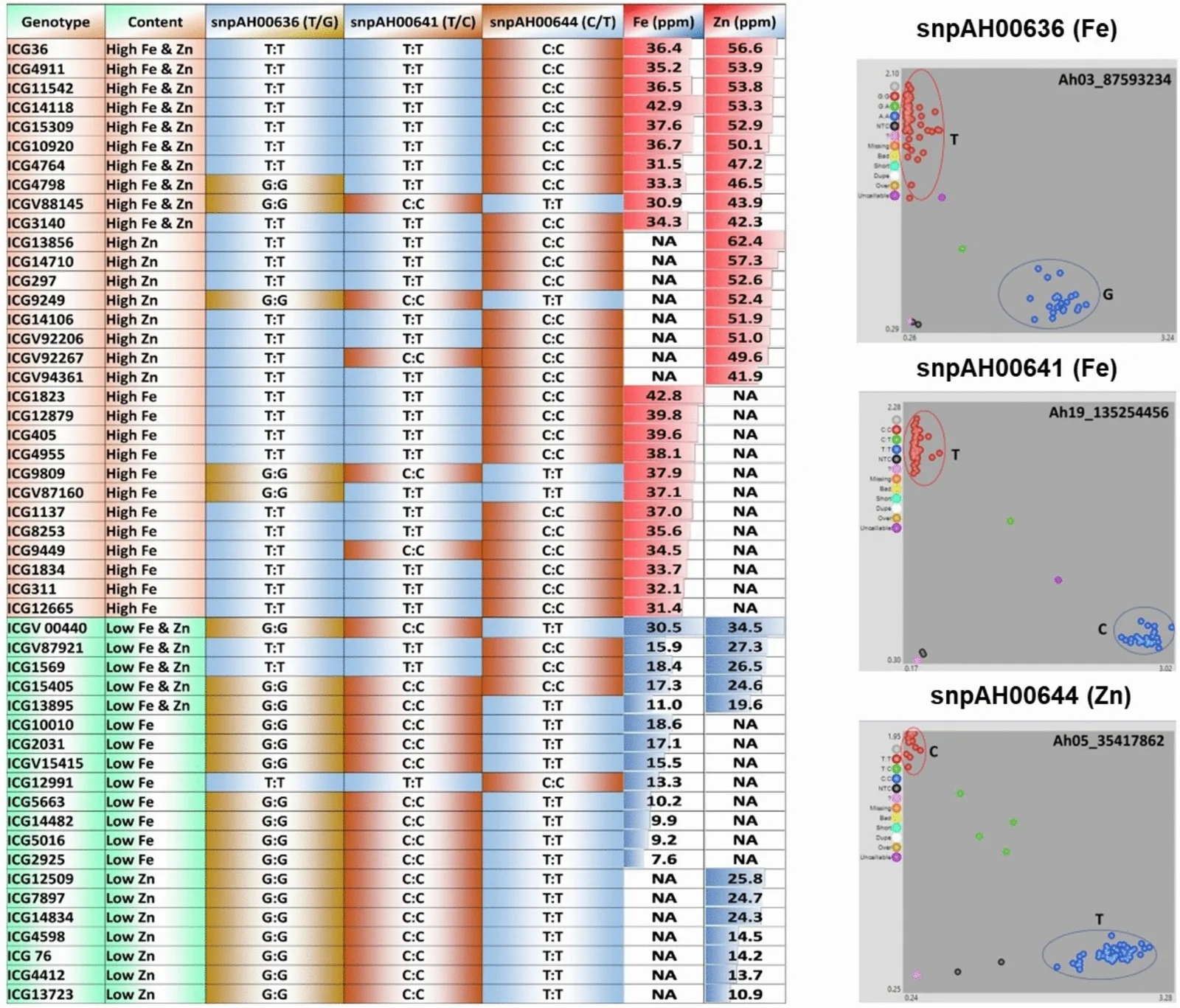
Validation of Kompetitive allele specific polymerase chain reaction (KASP) markers for Fe and Zn. KASP markers were validated in a panel consisting of 50 genotypes of both high and low Fe, Zn content lines with Fe ranging from 7.6 to 42.9 ppm and Zn ranging from 10.9 to 62.4 ppm. Three KASP markers (snpAH00636, snpAH00641 and snpAH00644) were successfully validated and showed polymorphism between high and low Fe, Zn content genotypes. SNPs show clear homozygous clusters- snpAH00636 for genes *Arahy.HNJF4G* (*26S proteasome regulatory subunit*), *Arahy.9RX38M* (*Development and Cell Death domain protein*); snpAH00641 for genes *Arahy.2U0JEX* (*UDP-Glycosyltransferase superfamily protein*), *Arahy.999YA8* (*calcium-dependent lipid-binding family protein*); snpAH00644 for genes *Arahy.AN6HNQ* (*uncharacterized protein*), *Arahy.GRNJ5W* (*Zinc finger, RING/FYVE/PHD-type*).

Based on the identified SNP polymorphisms, 9 MTAs (2 MTAs on Ah05, 2 MTAs on Ah18, 2 MTAs on Ah19, 1 MTA each on Ah03, Ah14 and Ah20) were used to develop KASP markers. These markers were successfully developed and validated in a panel of 50 genotypes, representing the ICRISAT reference set and mini-core collection (Fig. 4). The validation panel comprised of wide range of Fe (7.6 to 42.9 ppm) and Zn (10.9 to 62.4 ppm) contents. Of the nine validated KASPs (eight for Fe and one for Zn), three KASPs (snpAH00636—intergenic SNP between *Arahy.HNJF4G* and *Arahy.9RX38M* on chromosome Ah03, snpAH00641 -intergenic SNP between *Arahy.2U0JEX* and *Arahy.999YA8* on chromosome Ah19, snpAH00644—intergenic SNP between *Arahy.AN6HNQ* and *Arahy.GRNJ5W* on chromosome Ah05) showed clear polymorphism in the diverse germplasm. These 3 KASP markers can be used as diagnostic markers for their effective utilization in marker-assisted breeding for enhancing Fe and Zn in groundnut.

### Haplo-pheno analysis for the identification of superior haplotypes for Fe, Zn and PC

Haplo-pheno analysis with haplotype and phenotype data on 178 accessions was used to identify the superior haplotypes. The performance of a haplogroup (a set of genotypes with the same haplotype sequence) was compared to other haplogroups in order to identify a superior haplotype for the MTAs associated with Fe, Zn and PC. The 9 superior haplotypes for Fe were identified as H3 for Ah03_8759323, H4 for Ah18_55925435, H1 for Ah13_58751943, H1 for Ah17_12433761, H2 for Ah19_145627456, H4 for Ah13_17399102, H1 for Ah20_80483965, H4 for Ah09_10736323 and H1 for Ah19_13525456 (Supplementary Fig. 9 and 10). The 3 superior haplotypes for Zn were found to be H4 for Ah16_114034439, H2 for Ah05_15332507 and H4 for Ah05_35417862 (Supplementary Fig. 11). The superior haplotypes for PC were observed as H2 for Ah15_49814667 and H4 for Ah13_18601962 (Supplementary Fig. 12).

The superior haplotypes for the two traits- Fe and Zn majorly belong to the subspecies *fastigiata*, and agronomic type, Valencia bunch/Spanish bunch, whereas the inferior haplotypes of these traits belong to the subspecies *hypogaea*, and agronomic type, Virginia bunch/Virginia Runner. Similarly, the superior haplotypes related to the PC were observed in the accessions that belong to Spanish Bunch and Valencia Bunch, however the common accessions for the haplotypes with lowest mean values of PC were identified in all the agronomic types, Spanish Bunch/Valencia Bunch/Virginia Bunch/Virginia runner. Therefore, the haplotype diversity for PC did not show subspecies relationship with the inferior haplotype groups.

The genotypes that had superior haplotype combinations showed an increase in Fe and Zn levels in the accessions (ICG 14118, ICG 5494), compared to the accessions carrying inferior haplotype combinations (ICG 721, ICG 6813) which exhibited reduced levels of Fe and Zn. The genotypes ICG 14118, ICG 11249, ICG 9507, ICG 7906 and ICG 8567 exhibited increased Fe, Zn and PC levels with different combinations of superior haplotypes for the three traits (Supplementary Table 6).

## Discussion

Malnutrition, or “hidden hunger,” remains a significant global health challenge arising from inadequate nutrient intake. Biofortification offers a sustainable solution by improving essential nutritional content of crops, thus directly linking agriculture with nutrition and health^41^. Several crop varieties have been enriched with essential nutrients to combat malnutrition^41^, including groundnut, which provides essential nutrients and amino acids necessary for good health. Further enhancement of its protein and micronutrient content could make groundnut an important food crop in addressing malnutrition. The identification and characterization of genomic regions and candidate genes associated with these traits will advance biofortification efforts in groundnut.

In this study, significant phenotypic variability was observed for the three traits, consistent with earlier reports for Fe, Zn and PC in groundnut^42^, and other crops, such as chickpea^43^ and wheat^44^. Breeding strategies, such as multiple trait pyramiding, can be used to improve correlated traits like Fe and Zn^5^. A significant positive correlation was observed between Fe, Zn and PC across locations and seasons in this study. Similar results were previously reported in groundnut (*r* = 0.31)^5^, (*r* = 0.535)^42^, (*r* = 0.714)^26^ and in other legumes, including soybean (*r* = 0.29)^45^, mung bean (*r* = 0.469)^46^, and common bean (*r* = 0.416)^47^. These findings indicate that several common pathways are associated with the homeostasis of Fe and Zn. PCA further supported this, as Fe and Zn contributed positively to PCA1, while PC contributed to both PCA1 and PCA2. Similar results were observed in chickpea, where Fe and Zn loaded on PCA1^48^ and PC contributed to both PCA1 and PCA2^49^. This study highlights the genetic potential for enhancing Fe, Zn, and protein content in groundnut; however, the lack of multi-environment phenotypic data limited the ability to assess genotype stability. Micronutrient traits, including Fe and Zn, were significantly influenced by genotype-by-environment (G × E) interactions, and exhibited significant variability across different environments. Therefore, future studies should incorporate multi-environmental trials to validate these findings to ensure accurately capture environmental variance.

GWAS analysis detected a stable MTA (Ah03_28583444) on chromosome Ah03 for both Fe and Zn in S2. Similarly, a previous study identified a co-localized QTL linked with Fe and Zn on Ah03^5^. The genetic marker Ah03_28583444, closely associated with the MTA on chromosome Ah03, showed potential for further validation across diverse populations and could be used for early-generation selection in breeding programmes^50^. Previous studies reported significant QTLs for Fe, Zn on chromosomes Ah03, Ah05 and Ah13^5^, and for PC on chromosomes Ah03, Ah11, Ah12, and Ah18^51^, which are consistent with the present results. The highest number of MTAs for the three traits were identified on chromosomes Ah03, Ah17, Ah05 and Ah19. These genomic regions can be considered as hotspot regions for simultaneous trait improvement^52^. Another stable MTA (Ah03_125540699) associated with PC, was identified on Ah03 in four different seasons (S1, S2 at L1 and S1, S2 at L2), while two stable MTAs (Ah03_107178229, Ah03_116861708) on chromosome Ah03 were consistently detected in two different seasons (S2 at L2, S2 at L1). Earlier studies suggest that significant MTAs are less influenced by environmental variation^53^.

**Fig. 5:**
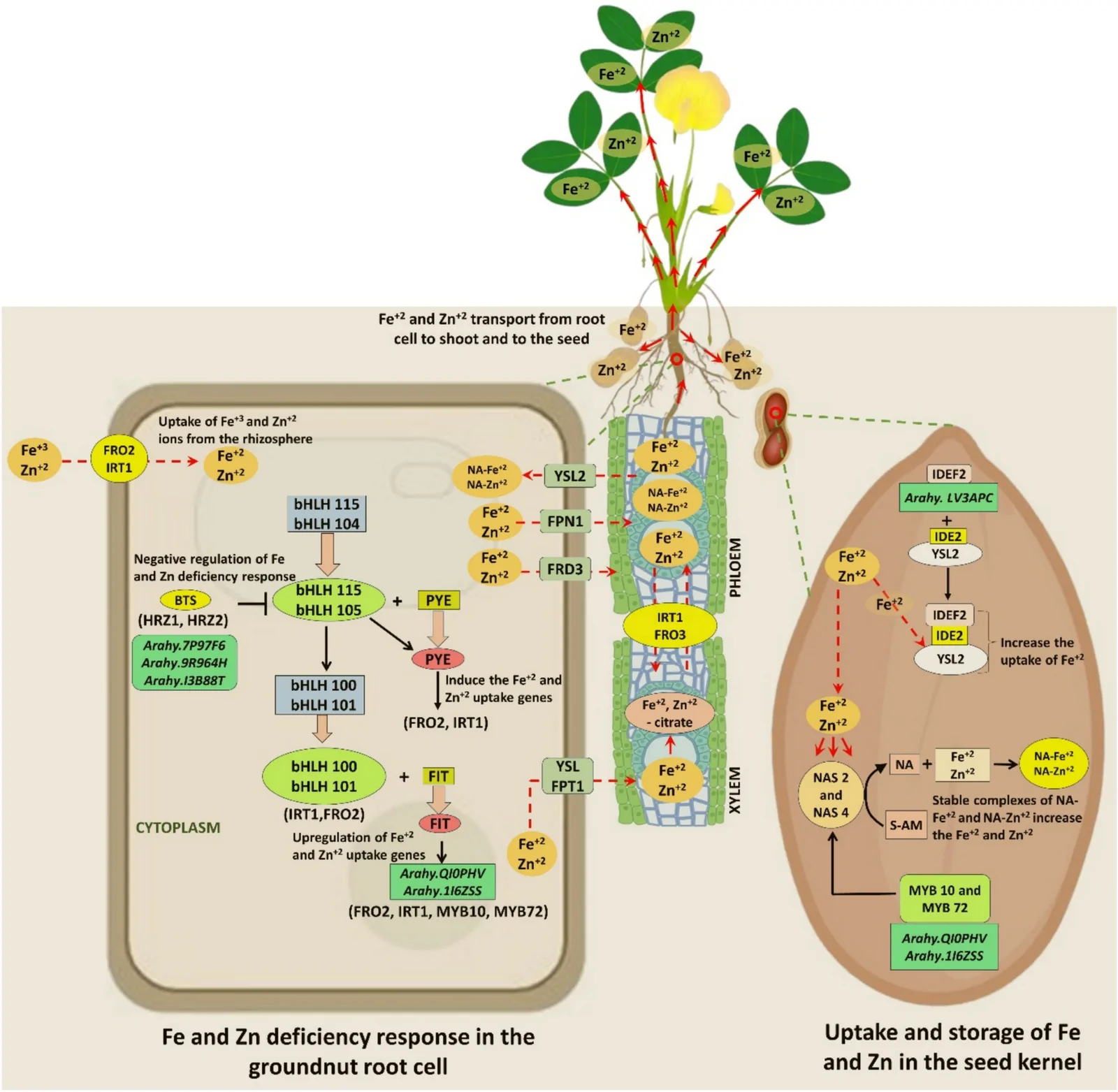
Molecular mechanism of Fe and Zn homeostasis in groundnut root cell and kernel. The figure depicts the Fe and Zn deficiency response in the groundnut root cell and the uptake, storage of both Fe and Zn in the groundnut kernel. *bHLH* basic helix-loop helix; *FIT* FER-like iron deficiency induced transcription factor; *FRO* ferric reduction oxidase; *IRT* iron-regulated transporter; *IRO* iron reduction oxidase; *MYB* myeoloblastosis; *PYE* POPEYE transcription factor; *NAS* nicotianamine adenine synthase; *HRZ* Haemerythrin motif-containing Really Interesting New Gene (RING)-and Zinc-finger protein; *IDEF* iron deficiency responsive element binding factor; *NA* nicotianamine adenine; *YSL* yellow stripe like; *VIT* vacuolar iron transporter; *NRAMP* natural resistance associated macrophage protein; *FRD* ferric reductase defective; *FPN* ferriportin; *IDE* iron deficiency responsive element; *SAM* S-adenosyl L-methionine.

This study identified key candidate genes associated with the significant SNPs that regulate the Fe and Zn homeostasis pathways. The genes encoding the proteins and transcription factors associated with Fe and Zn were found to suppress or inhibit the plant’s response to the deficiency conditions and also help in activation of a series of response mechanisms to enhance Fe and Zn uptake and homeostasis^54,55^ (Fig. 5). These genes facilitated Fe^+3^ and Zn^+2^ ions uptake from the rhizosphere into the root tissues and also involve in the mobilization of these ions from root to the shoot and seed^54,55^. The genes, *RING/ Zn finger domains* (*Arahy.7P97F6, Arahy.9R964H* and *Arahy.I3B88T*), which act as *E3 ubiquitin ligases* were identified to negatively regulate the Fe deficiency response^56,57^. Similarly, *MYB 10* and *MYB 72* that belong to the family, *MYB transcription factor* (*Arahy.QI0PHV, Arahy.1I6ZSS*), induce the *NAS* genes under the Fe and Zn deficiencies that increase the levels of Fe and Zn in the shoot and the seed^58^. Another transcription factor, *IDEF2* that belongs to the *NAC domain protein* family (*Arahy.LV3APC*), binds to cis-regulatory element, IDE2 present at the promoter site of Fe-deficiency response genes and controls the homeostasis of Fe by modulating the expression of these genes^59^. These genes serve as a valuable resource for the development of molecular markers and for further genetic and functional characterization^60^. The genes *MYB transcription factor* (*Arahy.QI0PHV*, *Arahy.1I6ZSS*), and *NAC domain* (*Arahy.LV3APC*) were earlier reported to show their expression in groundnut kernel^61,62^.

This study also reported several other candidate genes that are either directly or indirectly associated with the functional regulation of Fe, Zn and PC in the plants. Previous studies have shown that *P-loop containing nucleoside triphosphate hydrolases* (*Arahy.BEB4AA*) play a key role in the nuclear and cytosolic compartmentalisation, storage, and export of Fe in the forms of ferritin and ferroportin, and are involved in Zn-ion binding^57^. The gene*, ATP-binding/protein serine/threonine kinase* (*Arahy.11RBMI, Arahy.VMLN7L*, *Arahy.B3BEZ6*, and *Arahy.0IU8HI*), is involved in the homeostasis of Fe in *Arabidopsis* and the transcriptional activity regulation^63^. The *autophagy-related protein* (*Arahy.M50AVM*) is involved in the intracellular utilisation of Zn and inhibits the accumulation of reactive oxygen species (ROS) caused by Zn deficiency^64^. *Calcium-dependent protein kinase* family helps in the stress responsive regulation of Zn^+2^ ions^65^. The gene, *E3 ubiquitin-protein ligase* (*Arahy*.*PE3CF6*), identifies the target proteins and has crucial role in protein degradation and is a major component of ubiquitination cascade during plant response to stress^66^. *Heat shock proteins* (*HSPs*) play an important role in protein folding, assembly, translocation, and degradation under stress and in several regular cellular activities^67^.

The gene expression analysis based on the AhGEA data showed that *NAC transcription factor* (*Arahy.LV3APC)* showed higher expression in multiple plant tissues, including seeds, roots, and nodules. Whereas *MYB transcription factor* (*Arahy.QI0PHV*) exhibited higher expression in seeds, cotyledon and embryo, Earlier studies revealed the higher expression of *NAC genes* in root tissues, nodules, and developing seed^9,62^, and *MYB transcription factor* expressed highly in developing seed and shell^13,61^. Overall, this study highlights the crucial role of key candidate genes in Fe, Zn, and protein homeostasis. Identification and cloning of these genes will improve understanding of genetic and biological functioning of the traits. Furthermore, haplotype-based approaches using diverse sequencing data can aid in developing diagnostic markers to accelerate nutritional biofortification in groundnut.

Diagnostic markers enhance breeding efficiency by enabling early-generation selection through MAS^68^. The validated KASP markers for various traits, such as LLS, high oleic acid and leaf rust resistance, were widely utilized in groundnut breeding programs globally, contributing to the development of advanced breeding lines, some of which are available for commercial cultivation^69^. To identify and confirm the diagnostic markers associated with Fe and Zn, 9 SNPs were selected for the development of KASP markers, Among the 9 KASPs, 3 were successfully validated, and these validated markers can accelerate biofortification breeding programs through MAS.

LD based haplotype identification offers advantage, focusing on identifying comprehensive historical recombination events within a population and allowing for easy visualization^70^. Haplotypes offer a more comprehensive view by combining SNPs and other associated polymorphisms into inherited units^71^. Likewise, significant haplotype diversity was observed for the MTAs associated with the Fe, Zn and PC in the current study. Previous studies also reported superior haplotypes for Fe, Zn and PC in rice, wheat, sorghum and pigeonpea^72–74^. The different combinations of haplotypes showed variations in Fe and Zn contents, with superior haplotype combination showing higher levels of Fe and Zn. Therefore, pyramiding of superior haplotype blocks associated with micronutrient accumulation, using HBB, could enhance the concentrations of essential micronutrient elements, in the grains, including Zn^75,76^. In the present study, the accessions with superior haplotypes identified for Fe, Zn and PC showed better phenotypic performance compared to the accessions with inferior haplotypes (Supplementary Fig. 13). Overall, incorporation of these superior haplotypes in groundnut breeding programs has the potential for developing groundnut cultivars with improved kernel Fe, Zn, and PC (Supplementary Fig. 14).

## Conclusion

This study presents a comprehensive GWAS analysis utilizing WGRS data and multi-season phenotypic data from a mini-core collection to identify significant MTAs and candidate genes associated with Fe, Zn and PC in groundnut. A total of 15 MTAs and 28 candidate genes were identified from pooled season data, while 44 MTAs and 62 candidate genes were detected from seasonal data for Fe, Zn and PC. Among the nine KASP markers, three KASP markers (snpAH00636—intergenic SNP between *Arahy.HNJF4G* and *Arahy.9RX38M* on chromosome Ah03, snpAH00641—intergenic SNP between *Arahy.2U0JEX* and *Arahy.999YA8* on chromosome Ah19, snpAH00644—intergenic SNP between *Arahy.AN6HNQ* and *Arahy.GRNJ5W* on chromosome Ah05) showed clear polymorphism between high and low content genotypes in the diverse germplasm. Key candidate genes including *MYB transcription factor* (*Arahy.QI0PHV, Arahy.1I6ZSS*), *Zn finger MYM type protein* and *RING finger MYM type protein* (*Arahy.7P97F6*, *Arahy.9R964H* and *Arahy.I3B88T*), and *NAC domain protein* (*Arahy.LV3APC*) were found to be involved in the regulation of Fe and Zn homeostasis pathways. Haplotype analysis identified 9 superior haplotypes for Fe, 3 for Zn and 2 for PC. The haplo-pheno analysis revealed that superior haplotypes majorly found in the subspecies *fastigiata*, and agronomic type, Valencia bunch/Spanish bunch, whereas the haplotypes with the lowest mean values of these traits belong to the subspecies *hypogaea*, and agronomic type, Virginia bunch/Virginia Runner. The accessions ICG 14118, ICG 11249, ICG 9507, ICG 7906 and ICG 8567 showed higher contents of Fe, Zn and PC with different combinations of superior haplotypes. The superior haplotypes identified in the groundnut germplasm collection will serve as a potential resource for HBB for enhancing nutritional quality in groundnut.

## Supplementary Information


Supplementary Information 1.
Supplementary Information 2.
Supplementary Information 3.


## Data Availability

The data used in this study correspond to previously reported NCBI bio-projects ID (PRJNA1002116, PRJNA490835 and PRJNA490832).

## References

[1] Nwokolo, E. Peanut (*Arachis hypogaea* L.). *Food and Feed from Legumes and Oilseeds* 49–63 (Springer US, 1996).

[2] Krapovickas, A. & Gregory, W. C. Taxonomia Del Genero" Arachis (Leguminosae). *Bonplandia* 1–186 (1994).

[3] Stalker, T. & Wilson, R. F. eds. *Peanuts: Genetics, Processing, and Utilization* (Elsevier, 2015).

[4] Food and Agriculture Organization (FAO) (2023) [https://www.fao.org/faostat/en/#data]

[5] Parmar, S. et al. Genetic mapping identified major main-effect and three co-localized quantitative trait loci controlling high iron and zinc content in groundnut. *Plant Genome.* **16**, e20361 (2023). 37408143 10.1002/tpg2.20361 PMC12807354

[6] Organization, W. H. *World Health Statistics, [OP]: Monitoring Health for the Sustainable Development Goals* (World Health Organization, 2016).

[7] Parmar, S. et al. Recent advances in genetics, genomics, and breeding for nutritional quality in groundnut. *Accelerated Plant Breeding, Volume 4: Oil Crops* 111–137 (2022).

[8] World Health Organization. Community-based management of severe acute malnutrition: A joint statement by the World Health Organization, the World Food Programme, the United Nations System Standing Committee on Nutrition and the United Nations Children’s Fund. In *Community-based management of severe acute malnutrition: a joint statement by the World Health Organization, the World Food Programme, the United Nations System Standing Committee on Nutrition and the United Nations Children’s Fund*. (2007).

[9] Pandey, M. K. et al. Translational genomics for achieving higher genetic gains in groundnut. *Theor. Appl. Genet.* **133**, 1679–1702 (2020). 32328677 10.1007/s00122-020-03592-2 PMC7214508

[10] Pandey, M. K. et al. Development of a cost-effective high-throughput mid-density 5K genotyping assay for germplasm characterization and breeding in groundnut. *Plant Genome* **18**, e70019 (2025). 40164965 10.1002/tpg2.70019 PMC11958872

[11] Kadirimangalam, S. R., Bagudam, R., Variath, M. T. & Pasupuleti, J. Prospects of biofortification in groundnut using modern breeding approaches. In *Biofortification of Staple Crops* 345–377 (Springer Singapore, 2022).

[12] Gautam, A. et al. Fortifying crops with micronutrients for sustainable global nutritional security. *Agron. J.* **117**, e70086 (2025).

[13] Pandey, M. K. et al. Improved genetic map identified major QTLs for drought tolerance-and iron deficiency tolerance-related traits in groundnut. *Genes* **12**, 37 (2020). 33396649 10.3390/genes12010037 PMC7824586

[14] Xiong, H. et al. AhNRAMP1 iron transporter is involved in iron acquisition in peanut. *J. Exp. Bot.* **63**, 4437–4446 (2012). 22611231 10.1093/jxb/ers117 PMC3421984

[15] Anuradha, K. et al. Mapping QTLs and candidate genes for iron and zinc concentrations in unpolished rice of Madhukar× Swarna RILs. *Gene* **508**, 233–240 (2012). 22964359 10.1016/j.gene.2012.07.054

[16] Kaler, A. S., Gillman, J. D., Beissinger, T. & Purcell, L. C. Comparing different statistical models and multiple testing corrections for association mapping in soybean and maize. *Front. Plant Sci.* **10**, 1794 (2020). 32158452 10.3389/fpls.2019.01794 PMC7052329

[17] Price, A. L. et al. Principal components analysis corrects for stratification in genome-wide association studies. *Nat. Genet.* **38**, 904–909 (2006). 16862161 10.1038/ng1847

[18] Zhang, Y. M., Jia, Z. & Dunwell, J. M. The applications of new multi-locus GWAS methodologies in the genetic dissection of complex traits. *Front. Plant Sci.* **10**, 100 (2019). 30804969 10.3389/fpls.2019.00100 PMC6378272

[19] Segura, V. et al. An efficient multi-locus mixed-model approach for genome-wide association studies in structured populations. *Nat. Genet.* **44**, 825–830 (2012). 22706313 10.1038/ng.2314 PMC3386481

[20] Wang, J. & Zhang, Z. GAPIT version 3: Boosting power and accuracy for genomic association and prediction. *Genom. Proteomics Bioinform.* **19**, 629–640 (2021). 10.1016/j.gpb.2021.08.005 PMC9121400 34492338

[21] Lorenz, A. J., Hamblin, M. T. & Jannink, J. L. Performance of single nucleotide polymorphisms versus haplotypes for genome-wide association analysis in barley. *PLoS ONE* **5**, e14079 (2010). 21124933 10.1371/journal.pone.0014079 PMC2989918

[22] Contreras-Soto, R. I. et al. A genome-wide association study for agronomic traits in soybean using SNP markers and SNP-based haplotype analysis. *PLoS ONE* **12**, e0171105 (2017). 28152092 10.1371/journal.pone.0171105 PMC5289539

[23] Yan, J., Warburton, M. & Crouch, J. Association mapping for enhancing maize (*Zea mays* L.) genetic improvement. *Crop Sci.* **51**, 433–449 (2011).

[24] Qian, L. et al. Exploring and harnessing haplotype diversity to improve yield stability in crops. *Front. Plant Sci.* **8**, 1534 (2017). 28928764 10.3389/fpls.2017.01534 PMC5591830

[25] Upadhyaya, H. D., Bramel, P. J., Ortiz, R. & Singh, S. Developing a mini core of peanut for utilization of genetic resources. *Crop Sci.* **42**, 2150–2156 (2002).

[26] Upadhyaya, H. D., Dronavalli, N., Singh, S. & Dwivedi, S. L. Variability and stability for kernel iron and zinc contents in the ICRISAT mini core collection of peanut. *Crop Sci.* **52**, 2628–2637 (2012).

[27] Pandey, M. K. et al. Genomewide association studies for 50 agronomic traits in peanut using the ‘reference set’comprising 300 genotypes from 48 countries of the semi-arid tropics of the world. *PLoS ONE* **9**, e105228 (2014). 25140620 10.1371/journal.pone.0105228 PMC4139351

[28] Tang, D. et al. SRplot: A free online platform for data visualization and graphing. *PLoS ONE* **18**, e0294236 (2023). 37943830 10.1371/journal.pone.0294236 PMC10635526

[29] Kassambara, A. & Mundt, F. Factoextra: extract and visualize the results of multivariate data analyses. CRAN Contrib. Packag. (2016).

[30] Wheal, M. S., Fowles, T. O. & Palmer, L. T. A cost-effective acid digestion method using closed polypropylene tubes for inductively coupled plasma optical emission spectrometry (ICP-OES) analysis of plant essential elements. *Anal. Methods.* **3**, 2854–2863 (2011).

[31] Sharma, V. et al. Dissecting genomic regions and underlying candidate genes in groundnut MAGIC population for drought tolerance. *BMC Plant Biol.* **24**, 1044 (2024). 39497063 10.1186/s12870-024-05749-3 PMC11536578

[32] Chen, S., Zhou, Y., Chen, Y. & Gu, J. fastp: an ultra-fast all-in-one FASTQ preprocessor. *Bioinformatics* **34**, i884–i890 (2018). 30423086 10.1093/bioinformatics/bty560 PMC6129281

[33] Li, H. & Durbin, R. Fast and accurate short read alignment with Burrows-Wheeler transform. *Bioinformatics* **25**, 1754–1760 (2009). 19451168 10.1093/bioinformatics/btp324 PMC2705234

[34] Bertioli, D. J. et al. The genome sequence of segmental allotetraploid peanut *Arachis hypogaea*. *Nat. Genet.* **51**, 877–884 (2019). 31043755 10.1038/s41588-019-0405-z

[35] McKenna, A. et al. The Genome Analysis Toolkit: a MapReduce framework for analyzing next-generation DNA sequencing data. *Genome Res.* **20**, 1297–1303 (2010). 20644199 10.1101/gr.107524.110 PMC2928508

[36] Tang, Y. et al. GAPIT version 2: an enhanced integrated tool for genomic association and prediction. *Plant Geno.* **9**, 11 (2016). 10.3835/plantgenome2015.11.0120 27898829

[37] Alqudah, A. M., Sallam, A., Baenziger, P. S. & Börner, A. GWAS: fast-forwarding gene identification and characterization in temperate cereals: lessons from barley–a review. *J. Adv. Res.* **22**, 119–135 (2020). 31956447 10.1016/j.jare.2019.10.013 PMC6961222

[38] Sinha, P. et al. *Arachis hypogaea* gene expression atlas for *fastigiata* subspecies of cultivated groundnut to accelerate functional and translational genomics applications. *Plant Biotechnol. J.* **18**, 2187–2200 (2020). 32167667 10.1111/pbi.13374 PMC7589347

[39] He, C., Holme, J. & Anthony, J. SNP genotyping: The KASP assay. In *Crop Breeding: Methods and Protocols* 75–86 (Springer, 2014). 10.1007/978-1-4939-0446-4_7 24816661

[40] Milne, I. et al. Flapjack—Graphical genotype visualization. *Bioinformatics* **3134**, 3133–3134 (2010). 10.1093/bioinformatics/btq580 PMC2995120 20956241

[41] Jha, A. B. & Warkentin, T. D. Biofortification of pulse crops: Status and future perspectives. *Plants* **9**, 73 (2020). 31935879 10.3390/plants9010073 PMC7020478

[42] Janila, P. et al. Iron and zinc concentrations in peanut (*Arachis hypogaea* L.) seeds and their relationship with other nutritional and yield parameters. *J. Agric. Sci.* **153**, 975–994 (2015).

[43] Grewal, et al. Understanding genotypic variation and identification of promising genotypes for iron and zinc content in chickpea (*Cicer arietinum* L.). *J. Food Compos. Anal.* **103458**, 103458 (2020).

[44] Heidari, B., Padash, S. & Dadkhodaie, A. Variations in micronutrients, bread quality and agronomic traits of wheat landrace varieties and commercial cultivars. *Aust. J. Crop Sci.* **10**, 377–384 (2016).

[45] Wang, H. et al. Identification of quantitative trait loci (QTLs) and candidate genes of seed Iron and zinc content in soybean [*Glycine max* (L.) Merr.]. *BMC Genom.* **23**, 146 (2022). 10.1186/s12864-022-08313-1 PMC8857819 35183125

[46] Singh, R., Van Heusden, A. W., Kumar, R. & Visser, R. G. Genetic variation and correlation studies between micronutrient (Fe and Zn), protein content and yield attributing traits in mungbean (*Vigna. radiata* L.) Legum. *Res. Int. J.* **41**, 167–174 (2018).

[47] Tryphone, G. M. & Nchimbi-Msolla, S. Diversity of common bean (*Phaseolus vulgaris* L.) genotypes in iron and zinc contents under screen house conditions. *Afr. J. Agric. Res.* **5**, 738–747 (2010).

[48] Sukrutha, B., Reddy, C. K. K., Madhuri, K. N. & Reddy, C. B. R. Principal component analysis and path coefficient analysis for groundnut yield and seed quality attributes (*Arachis hypoga*ea L.) Legum. *Res. Int. J.* **48**, 1096–1102 (2025).

[49] Samineni, S. et al. Impact of heat and drought stresses on grain nutrient content in chickpea: Genome-wide marker-trait associations for protein, Fe and Zn. *Environ. Exp. Bot.* **194**, 104688 (2022).

[50] Roorkiwal, M. et al. Genome-wide association mapping of nutritional traits for designing superior chickpea varieties. *Front. Plant Sci.* **13**, 843911 (2022). 36082300 10.3389/fpls.2022.843911 PMC9445663

[51] Jadhav, M. P. et al. Genotyping-by-sequencing based genetic mapping identified major and consistent genomic regions for productivity and quality traits in peanut Front. *Plant Sci.* **12**, 668020 (2021). 10.3389/fpls.2021.668020 PMC8495222 34630444

[52] Sharma, V. et al. Genetic mapping identified three hotspot genomic regions and candidate genes controlling heat tolerance-related traits in groundnut. *Front. Plant Sci.* **14**, 1182867 (2023). 37287715 10.3389/fpls.2023.1182867 PMC10243373

[53] Sharma, V. et al. QTLs associated with yield attributing traits under drought stress in upland rice cultivar of Assam. *ORYZA-An Int. J. Rice.* **54**, 253–257 (2017).

[54] Robinson, N. J., Procter, C. M., Connolly, E. L. & Guerinot, M. L. A ferric-chelate reductase for iron uptake from soils. *Nature* **397**, 694–697 (1999). 10067892 10.1038/17800

[55] Curie, C. et al. Metal movement within the plant: contribution of nicotianamine and yellow stripe 1-like transporters. *Ann. Bot.* **103**, 1–11 (2009). 18977764 10.1093/aob/mcn207 PMC2707284

[56] Selote, D., Samira, R., Matthiadis, A., Gillikin, J. W. & Long, T. A. Iron-binding E3 ligase mediates iron response in plants by targeting basic helix-loop-helix transcription factors. *Plant Physiol.* **167**, 273–286 (2015). 25452667 10.1104/pp.114.250837 PMC4281009

[57] Jadon, et al. Molecular mapping of biofortification traits in bread wheat (*Triticum aestivum* L.) using a high-density SNP based linkage map. *Genes* **221**, 221 (2023). 10.3390/genes14010221 PMC9859277 36672962

[58] Palmer, C. M., Hindt, M. N., Schmidt, H., Clemens, S. & Guerinot, M. L. MYB10 and MYB72 are required for growth under iron-limiting conditions. *PLoS Genet.* **9**, e1003953 (2013). 24278034 10.1371/journal.pgen.1003953 PMC3836873

[59] Ogo, Y. et al. A novel NAC transcription factor, IDEF2, that recognizes the iron deficiency-responsive element 2 regulates the genes involved in iron homeostasis in plants. *J. Biol. Chem.* **283**, 13407–13417 (2008). 18308732 10.1074/jbc.M708732200

[60] Kearsey, M. J. TPo. Q. T. L. The principles of QTL analysis (a minimal mathematics approach). *J. Exp. Bot.* **1623**, 1619–1623 (1998).

[61] Wu, Y. et al. Comparative transcriptomics analysis of developing peanut (*Arachis hypogaea* L.) pods reveals candidate genes affecting peanut seed size. *Front. Plant Sci.* **13**, 958808 (2022). 36172561 10.3389/fpls.2022.958808 PMC9511224

[62] Yuan, C. et al. Comprehensive genomic characterization of NAC transcription factor family and their response to salt and drought stress in peanut. *BMC Plant Biol.* **20**, 454 (2020). 33008287 10.1186/s12870-020-02678-9 PMC7532626

[63] Tomita, M., Tanaka, H. & Takahashi, K. ABA-induced serine/threonine protein kinase gene transcribed in rye (*Secale cereale* L.). *Cereal Res. Commun.* **49**, 21–30 (2021).

[64] Eguchi, M., Kimura, K., Makino, A. & Ishida, H. Autophagy is induced under Zn limitation and contributes to Zn-limited stress tolerance in Arabidopsis (*Arabidopsis thaliana*). *Soil Sci. Plant Nutr.* **63**, 342–350 (2017).

[65] Tian, Q., Zhang, X., Yang, A., Wang, T. & Zhang, W. H. CIPK23 is involved in iron acquisition of Arabidopsis by affecting ferric chelate reductase activity. *Plant Sci.* **246**, 70–79 (2016). 26993237 10.1016/j.plantsci.2016.01.010

[66] Serrano, I., Campos, L. & Rivas, S. Roles of E3 ubiquitin-ligases in nuclear protein homeostasis during plant stress responses. *Front. Plant Sci.* **9**, 139 (2018). 29472944 10.3389/fpls.2018.00139 PMC5809434

[67] Park, C. J. & Seo, Y. S. Heat shock proteins: a review of the molecular chaperones for plant immunity. *Plant Pathol. J.* **31**, 323 (2015). 26676169 10.5423/PPJ.RW.08.2015.0150 PMC4677741

[68] Parmar, S. et al. Single seed-based high-throughput genotyping and rapid generation advancement for accelerated groundnut genetics and breeding research. *Agronomy* **11**, 1226 (2021).

[69] Pandey, M. K. et al. High-throughput diagnostic markers for foliar fungal disease resistance and high oleic acid content in groundnut. *BMC Plant Biol.* **24**, 262 (2024). 38594614 10.1186/s12870-024-04987-9 PMC11005153

[70] Liu, Y. et al. Phenotype prediction and genome-wide association study using deep convolutional neural network of soybean. *Front. Genet.* **10**, 1091 (2019). 31824557 10.3389/fgene.2019.01091 PMC6883005

[71] Bhat, J. A., Yu, D., Bohra, A., Ganie, S. A. & Varshney, R. K. Features and applications of haplotypes in crop breeding. *Commun. Biol.* **4**, 1266 (2021). 34737387 10.1038/s42003-021-02782-y PMC8568931

[72] Jamedar, H. V. R. et al. Identification of superior haplotypes for seed protein content in pigeonpea (*Cajanus Cajan* L.). *J. Plant Biochem. Biotechnol.* **33**, 178–188 (2024).

[73] Rohilla, M., Mazumder, A., Chowdhury, D. & Bhardwaj, R. Understanding natural genetic variation for nutritional quality in grain and identification of superior haplotypes in deepwater rice genotypes of Assam, India. *Gene* **148801**, 148801 (2024). 10.1016/j.gene.2024.148801 39068998

[74] Thakur, N. R. et al. Genome-wide association study and expression of candidate genes for Fe and Zn concentration in sorghum grains. *Sci. Rep.* **14**, 12729 (2024). 38830906 10.1038/s41598-024-63308-0 PMC11148041

[75] Borrill, P. et al. Biofortification of wheat grain with iron and zinc: Integrating novel genomic resources and knowledge from model crops. *Front. Plant Sci.* **5**, 53 (2014). 24600464 10.3389/fpls.2014.00053 PMC3930855

[76] Cheema, S. A., ur Rehman, H., Kiran, A., Bashir, K. & Wakeel, A. Progress and prospects for micronutrient biofortification in rice/wheat. *Plant Micronutr. Use Effic.* **2018**, 261–278 (2018).

