# Supplementary material for: Whole genome sequencing-based multi-locus association mapping for kernel iron, zinc and protein content in groundnut
Source: Sci Rep. 2026 Jun 13;16:27035. doi: 10.1038/s41598-026-57575-2 (PMC13522511; doi:10.1038/s41598-026-57575-2)
Supplement: Supplementary file 1 [file 41598_2026_57575_MOESM1_ESM.docx]

**Supplementary Figure 1.** Manhattan plots showing significant MTAs for Fe across five different seasons. The horizontal dashed lines represent the significant threshold kept at Bonferroni corrected *p-value* of 2 × 10^-7^ and the vertical dashed lines represent the candidate genes at the significant MTAs associated with Fe.

**Supplementary Figure 2.** QQ plots for Fe across five different seasons, to compare expected versus observed -log10(*p*) values.

**Supplementary Figure 3.** Manhattan plots showing significant MTAs for Zn across four different seasons. The horizontal dashed lines represent the significant threshold kept at Bonferroni corrected *p-value* of 2 × 10^-7^ and the vertical dashed lines represent the candidate genes at the significant MTAs associated with Zn.

**Supplementary Figure 4.** QQ plots for Zn across four different seasons, to compare expected versus observed -log10(*p*) values.

**Supplementary Figure 5.** Manhattan plots showing significant MTAs for PC across four different seasons. The horizontal dashed lines represent the significant threshold kept at Bonferroni corrected *p-value* of 2 × 10^-7^ and the vertical dashed lines represent the candidate genes at the significant MTAs associated with PC.

**Supplementary Figure 6.** QQ plots for PC across four different seasons, to compare expected versus observed -log10(*p*) values.

**Supplementary Figure 7.** Allelic segregation of associated MTAs from the pooled data analysis and phenotypic variations of allelic differences of associated SNPs for (A) Fe, (B) Zn and (C) PC.

**Supplementary Figure 8.** Allelic segregation of associated MTAs from the seasonal data analysis and phenotypic variations of allelic differences of associated SNPs for (A) Fe and (B) Zn.

**Supplementary Figure 9**. Superior haplotypes for high Fe content in groundnut. **(A, B, C)** LD based haplotype clustering of the SNPs (Ah03_87593234, Ah18_55925435, Ah13_58751943) associated with the Fe content showing haplotype variation. The accessions containing superior haplotypes were majorly belonging to Spanish/Valencia bunch and the accessions with inferior haplotypes were predominant in Virginia Bunch/Virginia Runner. **(D, E, F)** Haplo-pheno analysis revealed that H3 (Ah03_87593234), H4 (Ah18_55925435) and H1 (Ah13_58751943) confers high Fe content, while H4 (Ah03_87593234), H3 (Ah18_55925435) and H2 (Ah13_58751943) for low Fe content. **(G, H, I)** Box plots showing phenotypic variations of allelic differences of associated SNPs for Fe.

**Supplementary Figure 10**. Superior haplotypes for high Fe content in groundnut. **(A, B, C, D, E, F)** LD based haplotype clustering of the SNPs (Ah19_135254456, Ah13_17399102, Ah20_80483965, Ah09_107364323, Ah17_12433761, Ah19_145627456) associated with the Fe content showing haplotype variation. The accessions containing superior haplotypes were majorly belonging to Spanish/Valencia bunch and the accessions with inferior haplotypes were predominant in Virginia Bunch/Virginia Runner. **(G, H, I, J, K, L)** Haplo-pheno analysis revealed that H1 (Ah19_135254456), H4 (Ah13_17399102), H1 (Ah20_80483965), H4 (Ah09_107364323), H1 (Ah17_12433761) and H2 (Ah19_145627456) confers high Fe content, while H2 (Ah19_135254456), H1 (Ah13_17399102), H3 (Ah20_80483965), H1 (Ah09_107364323), H2 (Ah17_12433761) and H1 (Ah19_145627456) for low Fe content. **(M, N, O, P, Q, R)** Box plots showing phenotypic variations of allelic differences of associated SNPs for Fe.

 **Supplementary Figure 11**. Superior haplotypes for high Zn content in groundnut. **(A, B, C)** LD based haplotype clustering of the SNPs (Ah05_35417862, Ah05_15332507, Ah16_114034439) associated with the Zn content showing haplotype variation. The accessions containing superior haplotypes were majorly belonging to Spanish/Valencia bunch and the accessions with inferior haplotypes were predominant in Virginia Bunch/Virginia Runner. **(D, E, F)** Haplo-pheno analysis revealed that H4 (Ah05_35417862), H2 (Ah05_15332507) and H4 (Ah16_114034439) confers high Zn content, while H1 (Ah05_35417862), H3 (Ah05_15332507) and H2 (Ah16_114034439) for low Zn content. **(G, H, I)** Box plots showing phenotypic variations of allelic differences of associated SNPs for Zn.

**Supplementary Figure 12**. Superior haplotypes for high PC in groundnut. **(A, B)** LD based haplotype clustering of the SNPs (Ah13_18601962, Ah15_49814667) associated with the PC showing haplotype variation. The accessions containing superior haplotypes were majorly belonging to Spanish/Valencia bunch and the accessions with inferior haplotypes were predominant in all the agronomic types. **(C, D)** Haplo-pheno analysis revealed that H4 (Ah13_18601962) and H2 (Ah15_49814667) confers high PC, while H1 (Ah13_18601962) and H4 (Ah15_49814667) for low PC. **(E, F)** Box plots showing phenotypic variations of allelic differences of associated SNPs for PC.

**Supplementary Figure 13**. Developing tailored groundnut with superior haplotypes for Fe, Zn and PC. (**A**) The most inferior haplotype combination for Fe, Zn and PC is H4 for Ah03_87593234 (Fe), H1 for Ah05_35417862 (Zn) and H1 for Ah13_18601962 (PC). (**B**) The most superior haplotype combination for Fe, Zn and PC is H3 for Ah03_87593234 (Fe), H4 for Ah05_35417862 (Zn) and H4 for Ah13_18601962. New breeding lines can be developed through HBB with the most superior haplotype combination.

**Supplementary Figure 14**. Schematic representation of haplotype and haplo-pheno analysis towards developing donor lines with superior haplotypes for Fe and Zn contents. High Fe/Zn-associated haplotypes (red star), are predominantly present in Spanish bunch and Valencia bunch groundnut types, whereas Virginia runner and Virginia bunch types commonly possess low Fe/Zn haplotypes (green star). The accessions containing superior haplotype combinations associated with high Fe/Zn contents can be utilized as donors in the HBB programme for the development of groundnut cultivars with enhanced nutritional value.

**Supplementary Table 2** Phenotypic data collected across the seasons and locations for Fe, Zn and PC

| Seasons  Traits | 2008 R | 2009 R | 2009-10 PR | 2010-11 PR | 2020 R | 2021 R |
| --- | --- | --- | --- | --- | --- | --- |
| Iron | - | L1 | L1 | L1 | L1 | L1 |
| Zinc | - | L1 | L1 | L1 | L1 | L1 |
| Protein | L1 & L2 | L1 & L2 | - | - | L1 | L1 |

L1: ICRISAT	L2: Dharwad	R: Rainy	PR: Post rainy

**Supplementary Table 3** Primer sequences for the three KASP markers developed for validation on diverse germplasm lines of groundnut

| **S. No.** | **Intertek ID** | **SNP name** | **Chromosome** | **Position** | **Reference allele** | **Alternate allele** | **Primer sequence** | **Primer sequence** |
| --- | --- | --- | --- | --- | --- | --- | --- | --- |
| 1 | snpAH00636 | Ah03_87593234 | Ah03 | 87593234 | T | G | Forward 1 | TGGGCCCCTTTGGGCCTCTTTTGTGGTCTTCGAGCTCTTGGAAAAGAGGTCGGTGGGGGACCAATCTTTCATAAGGTCGGTCCTTTTGTCTACTGCGAGTCTAACTCTGTAGCTCATGTCTGGGTATGAGCAGAGAGATTCACTCAAATTGAGGCACAAGGAAGGAAAGACAAGGAAGCTATCAAGGCTAGAATTGTATACTTGTGTAAGGAGATATGGAAGGAGAGAAACAAAGCAACTTTAAACAATGCAGAAGTAAATCTATATTCAGCTATCATAAGAGCGATAATCACATAAAAA(T) |
|  |  |  |  |  |  |  | Forward 2 | TGGGCCCCTTTGGGCCTCTTTTGTGGTCTTCGAGCTCTTGGAAAAGAGGTCGGTGGGGGACCAATCTTTCATAAGGTCGGTCCTTTTGTCTACTGCGAGTCTAACTCTGTAGCTCATGTCTGGGTATGAGCAGAGAGATTCACTCAAATTGAGGCACAAGGAAGGAAAGACAAGGAAGCTATCAAGGCTAGAATTGTATACTTGTGTAAGGAGATATGGAAGGAGAGAAACAAAGCAACTTTAAACAATGCAGAAGTAAATCTATATTCAGCTATCATAAGAGCGATAATCACATAAAAA(G) |
|  |  |  |  |  |  |  | Reverse | AATTCAAAAATGCAAATGAGTTAGAAGACACAAGGTAATTAAAGGGATCAAGTCAAAGGAGGTACAAAAAGGTTACCTGGAGACCCCCTCCAGGGGTGTGGATAAAGGCGAACCTAGATGCAACATTCTTAAAAGACACAGTTGAAGGAGCAATTAGAATTATAATAAGGGAAAATTTAGGAAGGTTGGTCACAGGAACTGCAGAGAAGATTAACACGTCTTCATGCTTGACTACAGAAACATGTTGATAAATCCATATTTTACGATGATTTTTGAGTTGAAAAGTATAGAATTTATCAA |
| 2 | snpAH00641 | Ah19_135254456 | Ah19 | 135254456 | T | C | Forward 1 | CCATAAAAAATAACCCCCATACAACTCTCTGTCACTAGGACCAAAGAAAGTATATTCAAGTATTGGGAAGAGAGACTTCAATTCATATGTGGGTAGAGCATTCAAATTCTCATTCCCGGGAGCTTGCATGCATAGAGTTGAAGGGACTTGTATGGTTACTCCTTAGTTATTGGAATGTGTAAGCATGTAGTGTGCATGGCTTCCTCCTTTGTTTGTGCATCTTTCTCTTCTTCCATGTGTGAGGTGGAAAGTTCTCTAATTTACTAGAGTATGAACTCCTTTGATTAATTTTCTCACATT(T) |
|  |  |  |  |  |  |  | Forward 2 | CCATAAAAAATAACCCCCATACAACTCTCTGTCACTAGGACCAAAGAAAGTATATTCAAGTATTGGGAAGAGAGACTTCAATTCATATGTGGGTAGAGCATTCAAATTCTCATTCCCGGGAGCTTGCATGCATAGAGTTGAAGGGACTTGTATGGTTACTCCTTAGTTATTGGAATGTGTAAGCATGTAGTGTGCATGGCTTCCTCCTTTGTTTGTGCATCTTTCTCTTCTTCCATGTGTGAGGTGGAAAGTTCTCTAATTTACTAGAGTATGAACTCCTTTGATTAATTTTCTCACATT(C) |
|  |  |  |  |  |  |  | Reverse | TTCCGTAAGATCTTGCATTGTATCTCTTGGAAAAGAATTTGCTTGTTTCTCTATCATATGCTTGGTAATCAATTCCACGTGTTTTTCTATATTTTTTATGCTACCTCAAGTGGGTAGAGTAAACTCAGCAATATTTTGAGTAAACTCAGCAAAAGTATTCTCTAGGTTTTTCAATGCCAGCTCAACAGATGATAGTTCTTAATAGAAATAAAGAGATGATGTTTTTTTTATAAGAATAAAAAGGCGATGGTTCCTGATATGAACAGAGAGCTGATGGTTCTTGGTATAAATAAGAAAATG |
| 3 | snpAH00644 | Ah05_35417862 | Ah05 | 35417862 | C | T | Forward 1 | TGTACGCTTTTTCTTCTTTTTACATACACTTCTACTTCTTTTTCTTCTTCTTTTCTTATTTTTCTCTTATCTTTTTTTTCATTATTGTCGCCGTCATCACTACTACCACCACCTCCTCCTCCCTTTCCTTCTTTTTTCATTTGAATTTCTTCTTCTCATTCATTTTCCTCCTCCTTCTCATCCATCTTCATCATCACTGTTATCGTTATTGTCATCATTGTCGTCTTCTTCTTATACGTATAATAACATTTCTGTGTTTTCTAGTATTTGTGTGCTAAATAACACCAGAACTACAATTAT(C) |
|  |  |  |  |  |  |  | Forward 2 | TGTACGCTTTTTCTTCTTTTTACATACACTTCTACTTCTTTTTCTTCTTCTTTTCTTATTTTTCTCTTATCTTTTTTTTCATTATTGTCGCCGTCATCACTACTACCACCACCTCCTCCTCCCTTTCCTTCTTTTTTCATTTGAATTTCTTCTTCTCATTCATTTTCCTCCTCCTTCTCATCCATCTTCATCATCACTGTTATCGTTATTGTCATCATTGTCGTCTTCTTCTTATACGTATAATAACATTTCTGTGTTTTCTAGTATTTGTGTGCTAAATAACACCAGAACTACAATTAT(T) |
|  |  |  |  |  |  |  | Reverse | ATGCATGAACTAATTACACCAAATCCAAGTCAATGAGACTAAATGTACTATATTTAAATTCAAAATGCATCGAAATTACTTAATGATGACGACGCACAAATAAATTTAGTTCAAAACAAGTACAAACCAAGTACACCTTATTTAAATTCAGAATTCAGCGAAATTAATTAATGATGCCGACACACAAGCAAACTCAGTTCAAAACAAGCACATAAACTAGATTCAATCATCCACAAAAATACATACAAGTAAATTTTAAATCCAGAAAAGTCATTAATATGACTTAAATGACACCAGAAA |
| 4 | snpAH00637 | Ah05_10145205 | Ah05 | 10145205 | G | A | Forward 1 | TTTGTCCTTGTAATTTTTGAAGTTGATATTGTTTATGTTAGTCAGTAGAGATGTTGCAGTGTGAACTATAAATAGTACCGATATACAATACTAGCATCTCAGCTTGCTAGTTTGAACAAAATTCTTGAGTTTCAGTATTTCATAATTATCTATGTTTTATTAATTTTTGATGATGTCTTGGTTCATTTCAGGGTTGCAAAAGCTTTCCTTACTAAACCTGGAAGGGTGCCTTGTAACAGCTGCATGCTTGGATTCTCTTGTAGGTTTAATTGCTTGATATACTTTTATGCTTATTATGTT(G) |
|  |  |  |  |  |  |  | Forward 2 | TTTGTCCTTGTAATTTTTGAAGTTGATATTGTTTATGTTAGTCAGTAGAGATGTTGCAGTGTGAACTATAAATAGTACCGATATACAATACTAGCATCTCAGCTTGCTAGTTTGAACAAAATTCTTGAGTTTCAGTATTTCATAATTATCTATGTTTTATTAATTTTTGATGATGTCTTGGTTCATTTCAGGGTTGCAAAAGCTTTCCTTACTAAACCTGGAAGGGTGCCTTGTAACAGCTGCATGCTTGGATTCTCTTGTAGGTTTAATTGCTTGATATACTTTTATGCTTATTATGTT(A) |
|  |  |  |  |  |  |  | Reverse | CTGATCTAGAAAATTGGTCGCTTTATTATATTCCCTAGAAACAATACAACAATACAACTACTAAACTTATAAACCAGCCAATATATTTAAAGATATCCTTCAGTGGTGCCATTGTGAGAATGATTAAATTCTTGTCTTCTCTCTCTGGAAAACTTAATTTGTACAATTTGTAGAGTTGAAGTTCAACAAAAGCAATAAAAAGTTGCCATAAACAAGAATAAGAAGAGAAAAAGATGACACTTTTATTTGCAAAATAAATAACTTGAGTGGTTTATACACTCTGCTGTACAAATGTTATCC |
| 5 | snpAH00638 | Ah18_122103781 | Ah18 | 122103781 | T | C | Forward 1 | ATAATAGAAAGATAAAAAAATAGAGAATGAGAGAAAAGAGAGAAAGATAAAGATGAATAATGAGAGTGAGAATGAGAATTTGTTAATTTTGAAAGAAAAAATTTTATTTTAATTATAATAAAAAAATATCAGATGACATATTTTAATTTGTCAAATTACTAATATAAAATATAAATTATATATAGAATAAAAATAGTAGAGAGAAATAGAAAAATAGAGAGAGGTAGATGAGAGAATTTAGGAATGGAGTTTATTAATTTTGGAAAAAGATATTTTTTCTCAATTTTAATAAGAGAGTGT(T) |
|  |  |  |  |  |  |  | Forward 2 | ATAATAGAAAGATAAAAAAATAGAGAATGAGAGAAAAGAGAGAAAGATAAAGATGAATAATGAGAGTGAGAATGAGAATTTGTTAATTTTGAAAGAAAAAATTTTATTTTAATTATAATAAAAAAATATCAGATGACATATTTTAATTTGTCAAATTACTAATATAAAATATAAATTATATATAGAATAAAAATAGTAGAGAGAAATAGAAAAATAGAGAGAGGTAGATGAGAGAATTTAGGAATGGAGTTTATTAATTTTGGAAAAAGATATTTTTTCTCAATTTTAATAAGAGAGTGT(C) |
|  |  |  |  |  |  |  | Reverse | ATGCGACTCATTTGATTATTAAATTAGATAGTAATATATGATACATAATATAGGTATATTTCAATTTCAATATTTTAATTTTAATGCAATTAAAGAATGTCATGTTGCATATTCTGATTGTCAAATTAGTAATTAGTCATTGATATTAATATTAATATTAATAATAATATATAAAATAGATAGAGTGGTTGAAGGAATGAGAGAACTAGAGAAAAGAAGAGGGAGGAGGGAAGAATTCTTTAATTTTAGAGGAAAAGATTTGGTTTTAATTGTAATGAGAGAGTGACATGTGGCACATTT |
| 6 | snpAH00639 | Ah20_70265096 | Ah20 | 70265096 | C | G | Forward 1 | TCACTTTAATCCCATTCGATGCCTTGATATGTTTGTTGAGTAATTTTAGGTTCATAAGGCAAGTATTGGATGGAAGAAGTGAGGAGAAAAGCATGCAAAATGGGAGAACTCATGAAGAAATGAAGGAACCATAAAGTTATCAAGTCCGACCTCTTCGCACTCAATCGACCATAACTTGAGCTACAGAGGTCCAAATGAGGCGGTTCTAGTTGCGTTGGAAAGCTAACATCCGGGGCTTCGAAATGATATAAATTTTACTATATGTTGCTTCACGTTCAGGGGCGCGCACGCGCACTTTGC(C) |
|  |  |  |  |  |  |  | Forward 2 | TCACTTTAATCCCATTCGATGCCTTGATATGTTTGTTGAGTAATTTTAGGTTCATAAGGCAAGTATTGGATGGAAGAAGTGAGGAGAAAAGCATGCAAAATGGGAGAACTCATGAAGAAATGAAGGAACCATAAAGTTATCAAGTCCGACCTCTTCGCACTCAATCGACCATAACTTGAGCTACAGAGGTCCAAATGAGGCGGTTCTAGTTGCGTTGGAAAGCTAACATCCGGGGCTTCGAAATGATATAAATTTTACTATATGTTGCTTCACGTTCAGGGGCGCGCACGCGCACTTTGC(G) |
|  |  |  |  |  |  |  | Reverse | TGCGCGCGCCGATGCTGTCCGTGGCCCACCTTAATGAAATCGCCCCCAGCGATTTCTGAAGCACTTTGGGCCCAACCCAACTCATTTCTAATGCTATTCAAGCCAAGGATTGAAGGAGAGTCAACACACTTAGTCATAGTATAGTTTTTATCATGTTTTAGGCTAGAATTCTAGAGAGAGAGGCTCTCTCCTCTCTCTAGATTTAGGATAGAAATTAGGGTAGAGTTAAGTTAATCACTCTCAAATTTCTCTTTTAATTCTTGTTTTGAATTCAATTCTCTTTATGTTTCAATGTTCTAT |
| 7 | snpAH00640 | Ah18_55925435 | Ah18 | 55925435 | T | C | Forward 1 | TTTTAAAAATATTTTTCAAAAATCTTTTCTTAATTTTTTCTTATAATTTTCGAAAATATCATCAACAATTAATGTTTTGATTCAAAAATTTCAAGTTTGTTACTTACTTGTTAAGAAAGATTCAAACTTGAAGTTCTAGAATCATATCTTGTGATTTCTTGTGAATCAAGTCATTAATTGTGATTTAAAAATCAAATCTTTTTCAAAATTAATTTCAATCATATCTTTTCAAAAATATCTTTCTATCTTATCTTTTTCAAAATCATATCTTTTTAAAACATATCTTTTTCAAAAATTTGA(T) |
|  |  |  |  |  |  |  | Forward 2 | TTTTAAAAATATTTTTCAAAAATCTTTTCTTAATTTTTTCTTATAATTTTCGAAAATATCATCAACAATTAATGTTTTGATTCAAAAATTTCAAGTTTGTTACTTACTTGTTAAGAAAGATTCAAACTTGAAGTTCTAGAATCATATCTTGTGATTTCTTGTGAATCAAGTCATTAATTGTGATTTAAAAATCAAATCTTTTTCAAAATTAATTTCAATCATATCTTTTCAAAAATATCTTTCTATCTTATCTTTTTCAAAATCATATCTTTTTAAAACATATCTTTTTCAAAAATTTGA(C) |
|  |  |  |  |  |  |  | Reverse | TTTAAAATATCTTCTCTAACTTCTTATCTTCTTATCTTTTCAAAATTGATTTTCAAATTTGTTTCAACTAACTAACTAACTTTTTGTTTGTTTCTTATCTTTTTCAAAACTACCTAACTAACTCTCTCTCTCTAATTTTCGAAAATATCTCCCTCTTTTTCAAAAATTCTTTTTAATTAACTAATTGTTTCAATTTTTAATTTTAAACTTGATTTCATTCCTAATTTTCGAAAATCACTAACCTCTTTTCAAAATTTTATTTTTGAAAATTCTCTTTCTCTCTCATCTCCTTCTATTTAT |
| 8 | snpAH00642 | Ah19_145627456 | Ah19 | 145627456 | C | T | Forward 1 | TTCGAAAGCAATGAGTCTGAACTCTTTCAGCTCATTCAACTGGAGTAATCTTTTCTCTCCAGCTACCTTAGCATCAAGGTTTAGGAACCTGGTTGCCCAGTAGGCTTTGTGTTCTAGCTCCACTGGCAGATGACAGGCTTTGCCATACACAAGCTGGTATGGAGAGGTCCCTATAGGAGTCTTGAATGCTGTTCTGTATGCCCACAGAGCATCATCCAGGCTTCTTGCCCAGTCCTTTCTACGGATACTTACAGTCCGTTCCAGGATTCGTTTTAGTTCTCTATTCAAAACTTCAGCCTG(C) |
|  |  |  |  |  |  |  | Forward 2 | TTCGAAAGCAATGAGTCTGAACTCTTTCAGCTCATTCAACTGGAGTAATCTTTTCTCTCCAGCTACCTTAGCATCAAGGTTTAGGAACCTGGTTGCCCAGTAGGCTTTGTGTTCTAGCTCCACTGGCAGATGACAGGCTTTGCCATACACAAGCTGGTATGGAGAGGTCCCTATAGGAGTCTTGAATGCTGTTCTGTATGCCCACAGAGCATCATCCAGGCTTCTTGCCCAGTCCTTTCTACGGATACTTACAGTCCGTTCCAGGATTCGTTTTAGTTCTCTATTCAAAACTTCAGCCTG(T) |
|  |  |  |  |  |  |  | Reverse | CCATTTGTCTATGGATGATTTGGAGTCACCACTTTGTGGTTAATTCCATATTGGACCAGAGCAGAGTCAAGCTATTTATTGCAAAAATGAGTGCCTCCATCACTGATTAGTATCCTAGGGACACCGAACCTGCTGAAGATATGCTTCTGGAGGAACTTCATTACTATCTTAGTATCATTAGTGGGTGTTGCAATTGCCTCTACCCATTTAGATACATAGTCTACTGCCACCAGAATATAAGTGTTTAAGTATGATGGTGGGAAAGGCCCCATGAAGTTAATACTCCATACATCAAACAAC |
| 9 | snpAH00643 | S14_5692509 | Ah14 | 5692509 | A | G | Forward 1 | AAAATTATTTTAGTGACTAAATTTAATTAAATTGGTCTAATAATTTAAGAATTGATAATAAAAAATTTTAATTAAAAAAAGCAAAAAAAAAATAAAAAAGGTTTAATTAAATTTAATTTAAATAATAATTTCTTCGCCTACCATCTCTTTAATCCATATTTTTACACATAAATCACATATATCATGTGTATATAAAAGACTAATTATAGACACCAAAAAATATTTTTAAAAAACTAATTATTAAGTTATTGACTTTTCCTATTGTGCCAAGGAATTAAGTTATTAGTTGAATTTAAAATT(A) |
|  |  |  |  |  |  |  | Forward 2 | AAAATTATTTTAGTGACTAAATTTAATTAAATTGGTCTAATAATTTAAGAATTGATAATAAAAAATTTTAATTAAAAAAAGCAAAAAAAAAATAAAAAAGGTTTAATTAAATTTAATTTAAATAATAATTTCTTCGCCTACCATCTCTTTAATCCATATTTTTACACATAAATCACATATATCATGTGTATATAAAAGACTAATTATAGACACCAAAAAATATTTTTAAAAAACTAATTATTAAGTTATTGACTTTTCCTATTGTGCCAAGGAATTAAGTTATTAGTTGAATTTAAAATT(G) |
|  |  |  |  |  |  |  | Reverse | ATAAAAAAAATGTTTACAAATACTAAAAATTAGTTGGTATATTTATATATGTATTATTTTATATTTTTCGATAAAGTGAATAGCCATTAAAATTTAAAATAATCAAATTTTTTATAATAAAATACTCAATTTTTTTTATAAAATAATCAAACTTTTTCACGATGATGAAACCAAATGTTAATAAATACAAAAAATCTGTTGTCATTATATTTTTATATATGTTAAATTTCTGAGAAAAGAGCATAATAAGAAAGATAATATTATATGTAAAGTTGTTGTGTTGAAGTTGTTGTTATATAA |

**Supplementary Table 4** Details of the high and low Fe/Zn lines used for the validation of KASP markers

|  | **snpAH00636** | snpAH00637 | snpAH00638 | snpAH00639 | snpAH00640 | **snpAH00641** | snpAH00642 | snpAH00643 | **snpAH00644** |  |  |  |
| --- | --- | --- | --- | --- | --- | --- | --- | --- | --- | --- | --- | --- |
| Genotype | **Ah03_87593234 (T/G)** | Ah05_10145205 (G/A) | Ah18_122103781 (T/C) | Ah20_70265096 (C/G) | Ah18_55925435 (T/C) | **Ah19_135254456 (T/C)** | Ah19_145627456 (C/T) | Ah14_5692509 (A/G) | **Ah05_35417862 (C/T)** | Content | Fe content (ppm) | Zn content (ppm) |
| ICG10920 | T:T | G:G | T:T | G:G | T:T | T:T | C:C | A:A | C:C | High Fe & Zn | 36.7 | 50.1 |
| ICG11542 | T:T | A:G | T:T | G:G | T:T | T:T | C:C | A:A | C:C | High Fe & Zn | 36.5 | 53.8 |
| ICG14118 | T:T | A:G | T:T | G:G | T:T | T:T | C:C | A:A | C:C | High Fe & Zn | 42.9 | 53.3 |
| ICG15309 | T:T | A:G | T:T | G:G | T:T | T:T | C:C | A:A | C:C | High Fe & Zn | 37.6 | 52.9 |
| ICG3140 | T:T | A:G | T:T | G:G | T:T | T:T | C:C | A:A | C:C | High Fe & Zn | 34.3 | 42.3 |
| ICG36 | T:T | A:G | T:T | G:G | T:T | T:T | C:C | A:A | C:C | High Fe & Zn | 36.4 | 56.6 |
| ICG4764 | T:T | G:G | T:T | G:G | T:T | T:T | C:C | A:A | C:C | High Fe & Zn | 31.5 | 47.2 |
| ICG4798 | G:G | G:G | C:C | G:G | T:T | T:T | C:C | G:A | C:C | High Fe & Zn | 33.3 | 46.5 |
| ICG4911 | T:T | A:G | T:T | G:G | T:T | T:T | C:C | A:A | C:C | High Fe & Zn | 35.2 | 53.9 |
| ICGV88145 | G:G | A:G | C:C | G:G | T:T | C:C | T:T | A:A | T:T | High Fe & Zn | 30.9 | 43.9 |
| ICG13856 | T:T | G:G | T:T | G:G | T:T | T:T | C:C | A:A | C:C | High Zn | . | 62.4 |
| ICG14106 | T:T | G:G | T:T | G:G | T:T | T:T | C:C | A:A | C:C | High Zn | . | 51.9 |
| ICG14710 | T:T | G:G | T:T | G:G | T:T | T:T | C:C | A:A | C:C | High Zn | . | 57.3 |
| ICG297 | T:T | G:G | T:T | G:G | T:T | T:T | C:C | A:A | C:C | High Zn | . | 52.6 |
| ICG9249 | G:G | G:G | C:C | G:G | T:T | C:C | T:C | G:A | T:T | High Zn | . | 52.4 |
| ICGV92206 | T:T | A:G | T:T | G:G | T:T | T:T | C:C | A:A | C:C | High Zn | . | 51 |
| ICGV92267 | T:T | A:G | C:C | G:G | T:T | C:C | T:T | A:A | C:C | High Zn | . | 49.6 |
| ICGV94361 | T:T | A:G | T:T | G:G | T:T | T:T | C:C | . | C:C | High Zn | . | 41.9 |
| ICG1137 | T:T | A:G | T:T | G:G | T:T | T:T | C:C | A:A | C:C | High Fe | 37 | . |
| ICG12665 | T:T | G:G | T:T | G:G | T:T | T:T | C:C | A:A | C:C | High Fe | 31.4 | . |
| ICG12879 | T:T | A:G | T:T | G:G | T:T | T:T | C:C | A:A | C:C | High Fe | 39.8 | . |
| ICG1823 | T:T | A:G | T:T | G:G | T:T | T:T | C:C | A:A | C:C | High Fe | 42.8 | . |
| ICG1834 | T:T | A:G | T:T | G:G | T:T | T:T | C:C | A:A | C:C | High Fe | 33.7 | . |
| ICG311 | T:T | A:G | T:T | G:G | T:T | T:T | C:C | A:A | C:C | High Fe | 32.1 | . |
| ICG405 | T:T | G:G | T:T | G:G | T:T | T:T | C:C | A:A | C:C | High Fe | 39.6 | . |
| ICG4955 | T:T | A:G | T:T | G:G | T:T | T:T | C:C | A:A | C:C | High Fe | 38.1 | . |
| ICG8253 | T:T | A:G | T:T | G:G | T:T | T:T | C:C | A:A | C:C | High Fe | 35.6 | . |
| ICG9449 | T:T | A:G | T:T | G:G | T:T | C:C | T:T | . | C:C | High Fe | 34.5 | . |
| ICG9809 | G:G | G:G | C:C | G:G | T:T | C:C | T:C | G:A | T:T | High Fe | 37.9 | . |
| ICGV87160 | G:G | A:G | C:C | G:G | T:T | T:T | C:C | A:A | T:T | High Fe | 37.1 | . |
| ICGV00440 | G:G | G:G | C:C | G:G | T:T | C:C | T:T | . | T:T | Low Fe & Zn | 30.5 | 34.5 |
| ICG13895 | G:G | G:G | C:C | G:G | T:T | C:C | T:T | . | T:T | Low Fe & Zn | 11 | 19.6 |
| ICG15405 | G:G | A:G | T:T | G:G | T:T | C:C | T:T | . | C:C | Low Fe & Zn | 17.3 | 24.6 |
| ICG1569 | T:T | A:G | T:T | G:G | T:T | T:T | C:C | A:A | C:C | Low Fe & Zn | 18.4 | 26.5 |
| ICGV87921 | T:T | A:G | T:T | G:G | T:T | T:T | C:C | A:A | C:C | Low Fe & Zn | 15.9 | 27.3 |
| ICG10010 | G:G | G:G | T:T | G:G | T:T | C:C | C:C | . | T:T | Low Fe | 18.6 | . |
| ICG12991 | T:T | A:G | T:T | G:G | T:T | T:T | C:C | A:A | C:C | Low Fe | 13.3 | . |
| ICG14482 | G:G | . | C:C | G:G | T:T | C:C | T:C | G:A | T:T | Low Fe | 9.9 | . |
| ICGV15415 | G:G | . | . | G:G | . | C:C | T:T | G:A | T:T | Low Fe | 15.5 | . |
| ICG2031 | G:G | A:G | T:T | G:G | T:T | C:C | C:C | G:A | T:T | Low Fe | 17.1 | . |
| ICG2925 | G:G | G:G | C:C | G:G | T:T | C:C | T:T | G:A | T:T | Low Fe | 7.6 | . |
| ICG5016 | G:G | G:G | T:T | G:G | T:T | C:C | T:C | G:A | T:T | Low Fe | 9.2 | . |
| ICG5663 | G:G | A:G | C:C | G:G | T:T | C:C | T:C | G:A | T:T | Low Fe | 10.2 | . |
| ICG12509 | G:G | G:G | C:C | G:G | T:T | C:C | T:T | G:A | T:T | Low Zn | . | 25.8 |
| ICG13723 | G:G | G:G | C:C | G:G | T:T | C:C | T:T | G:A | T:T | Low Zn | . | 10.9 |
| ICG14834 | G:G | G:G | C:C | G:G | T:T | C:C | . | G:A | T:T | Low Zn | . | 24.3 |
| ICG4412 | G:G | G:G | C:C | G:G | T:T | C:C | T:T | G:A | T:T | Low Zn | . | 13.7 |
| ICG4598 | G:G | A:G | C:C | G:G | T:T | C:C | T:C | G:A | T:T | Low Zn | . | 14.5 |
| ICG76 | G:G | . | C:C | G:G | T:T | C:C | T:C | G:A | T:T | Low Zn | . | 14.2 |
| ICG7897 | G:G | G:G | C:C | G:G | T:T | C:C | T:T | . | T:T | Low Zn | . | 24.7 |

**Supplementary Table 6** Combining effect of the haplotypes for the MTAs associated with Fe, Zn and PC in the diverse groundnut accessions differentiating high and low Fe, Zn and PC across various agronomic types

| **Genotypes** | **Ah03_87593234 (Fe)** | **Ah18_55925435 (Fe)** | **Ah13_58751943 (Fe)** | **Ah19_135254456 (Fe)** | **Ah13_17399102 (Fe)** | **Ah20_80483965 (Fe)** | **Ah09_107364323 (Fe)** | **Ah17_12433761 (Fe)** | **Ah19_145627456 (Fe)** | **Ah05_35417862 (Zn)** | **Ah16_114034439 (Zn)** | **Ah05_15332507 (Zn)** | **Ah13_18601962 (PC)** | **Ah15_49814667 (PC)** | **Fe Mean (ppm)** | **Zn Mean (ppm)** | **PC Mean (%)** | **Sub Species** | **Agronomic type** |
| --- | --- | --- | --- | --- | --- | --- | --- | --- | --- | --- | --- | --- | --- | --- | --- | --- | --- | --- | --- |
| **High content genotypes** | | | | | | | | | | | | | | | | | | | |
| ICG9507 | . | H4* | . | H1* | H4* | H1* | H2 | . | H2* | H2 | H1 | H2* | . | H2* | 30.23 | 38.76 | 26.88 | *fastigiata* | Spanish Bunch |
| ICG8083 | . | . | . | . | . | . | . | H1* | . | . | . | H2* | H4* | . | 24.24 | 38.24 | 26.69 | *fastigiata* | Spanish Bunch |
| ICG7906 | . | . | . | . | H4* | . | . | . | . | . | . | H2* | H4* | . | 24.44 | 37.59 | 25.15 | *fastigiata* | Spanish Bunch |
| ICG3343 | H3* | H4* | H1* | . | . | . | . | . | H2* | H2 | H1 | . | H3 | . | 27.72 | 37 | 26.45 | *fastigiata* | Spanish Bunch |
| ICG1973 | . | . | . | H1* | . | H1* | H3 | . | H2* | H2 | H4* | . | H4* | . | 23.06 | 36.99 | 28.55 | *fastigiata* | Spanish Bunch |
| ICG14118 | . | . | . | . | . | . | . | H1* | . | H4* | . | H2* | H4* | . | 25.98 | 36.79 | 26.13 | *fastigiata* | Spanish Bunch |
| ICG8567 | H1 | . | . | . | . | H1* | . | . | H2* | H2 | H1 | H2* | H4* | . | 25.93 | 36.71 | 20.87 | *fastigiata* | Spanish Bunch |
| ICG13491 | . | H1 | H1* | H1* | H3 | . | . | . | H2* | . | . | . | H4* | . | 27.6 | 36.41 | 27.66 | *fastigiata* | Spanish Bunch |
| ICG3421 | . | H1 | H1* | H1* | H1 | . | H4* | H1* | H2* | H2 | H1 | H2* | H4* | . | 25.28 | 36.2 | 28.86 | *fastigiata* | Spanish Bunch |
| ICG6263 | H1 | . | H1* | H1* | H4* | . | H2 | H1* | H2* | . | H1 | . | H4* | . | 26.64 | 36.17 | 25.91 | *fastigiata* | Spanish Bunch |
| ICG11249 | . | H1 | . | . | . | . | . | H1* | . | . | . | H2* | H4* | . | 25.32 | 35.37 | 27.04 | *fastigiata* | Spanish Bunch |
| ICG3584 | . | H4* | H1* | H1* | H1 | . | . | . | H2* | H2 | H1 | . | . | . | 24.08 | 34.54 | 27.53 | *fastigiata* | Spanish Bunch |
| ICG4543 | . | . | H1* | H1* | H3 | H1* | H2 | H1* | H2* | H2 | H1 | H2* | H4* | H4 | 26.13 | 31.63 | 26.67 | *fastigiata* | Spanish Bunch |
| ICG2019 | . | . | . | H1* | . | H1* | H4* | . | . | . | . | . | . | . | 23.13 | 33.33 | 29.12 | *fastigiata* | Spanish Bunch |
| ICG1137 | . | H4* | . | . | . | . | . | H1* | H2* | . | . | . | . | . | 28.82 | 32.84 | 26.37 | *fastigiata* | Spanish Bunch |
| ICG12697 | . | . | H1* | . | . |  | . | H1* | H2* | . | . | . | . | . | 24.04 | 36.78 | 25.24 | *fastigiata* | Spanish Bunch |
| ICG13603 | . | . | . | H1* | . | H1* | . | H1* | H2* | . | . | . | . | H2* | 27.57 | 37.64 | 27.55 | *fastigiata* | Spanish Bunch |
| ICG4955 | . | . | . | H1* | . | H1* | . | H1* | H2* | . | . | . | . | . | 26.73 | 37.27 | 18.83 | *fastigiata* | Spanish Bunch |
| ICG1519 | . | . | . | . | . | . | . | H1* | H2* | . | . | . | . | . | 22.1 | 34.26 | 30.03 | *fastigiata* | Spanish Bunch |
| ICG36 | . | . | H1* | H1* | . | H1* | . | H1* | . | . | . | . | . | . | 26.41 | 38.01 | 28.92 | *fastigiata* | Spanish Bunch |
| ICG1274 | H1 | H4* | . | . | . | H1* | H3 | . | H3 | H2 | . | H2* | . | . | 27.42 | 41.06 | 23.48 | *fastigiata* | Valencia Bunch |
| ICG15309 | . | H4* | H1* | H1* | . | . | . | . | . | . | . | . | . | . | 29.37 | 37.41 | 18.49 | *fastigiata* | Valencia Bunch |
| ICG5494 | . | . | . | . | H1 | . | . | H1* | . | H4* | . | H2* | . | . | 28.28 | 36.26 | 26.68 | *fastigiata* | Valencia Bunch |
| ICG10554 | . | H4* | H1* | H3 | . | . | . | H3 | H2* | . | . | H5 | H4* | H4 | 26.87 | 31.96 | 27.18 | *fastigiata* | Valencia Bunch |
| ICG6888 | . | . | . | . | H4* | H1* | . | . | . | . | . | . | . | . | 25.43 | 37.56 | 28.55 | *fastigiata* | Valencia Bunch |
| ICG8106 | . | . | . | H1* | . | H1* | H4* | . | H2* | . | . | . | . | . | 25.31 | 33.25 | 21.77 | *fastigiata* | Valencia Bunch |
| ICG14710 | . | H4* | . | . | H4* | H1* | . | . | . | . | . | . | . | . | 26.67 | 40.74 | 23.41 | *hypogaea* | . |
| **Low content genotypes** | | | | | | | | | | | | | | | | | | | |
| ICG5662 | H4 | H3 | . | . | H1 | H3 | H1 | . | . | . | H2 | . | . | . | 24.53 | 39.01 | 26.15 | *hypogaea* | Virginia Bunch |
| ICG14466 | . | . | H2 | . | H1 | . | H1 | H2 | H1 | H1 | H2 | H1 | H1 | . | 21.6 | 34.13 | 24.93 | *hypogaea* | Virginia Bunch |
| ICG3053 | H4 | . | . | H2 | . | H3 | . | H2 | . | H1 | . | H1 | H1 | . | 21.53 | 33.12 | 24.16 | *hypogaea* | Virginia Bunch |
| ICG188 | H3* | H3 | H2 | . | H1 | H3 | . | H2 | H1 | . | . | . | . | . | 23.92 | 33.08 | 18.58 | *hypogaea* | Virginia Bunch |
| ICG3027 | . | H3 | . | . | . | . | H1 | . | H1 | H1 | H2 | H1 | H1 | H1 | 20.12 | 29.2 | 22.81 | *hypogaea* | Virginia Bunch |
| ICG12000 | H4 | . | . | H2 | H1 | H3 | H1 | H2 | H1 | H1 | . | H1 | H1 | H1 | 23.17 | 29.15 | 20.64 | *hypogaea* | Virginia Bunch |
| ICG13787 | H4 | . | . | H2 | H1 | H3 | H1 | . | H1 | . | H2 | . | H1 | . | 21.39 | 28.98 | 22.03 | *hypogaea* | Virginia Bunch |
| ICG11322 | H4 | . | . | . | H1 | H3 | H1 | H2 | . | H2 | H1 | H3 | H1 | . | 22.26 | 28.96 | 24.99 | *hypogaea* | Virginia Bunch |
| ICG513 | . | H3 | . | H2 | . | H3 | . | H2 | H1 | H1 | . | . | H1 | H1 | 19.99 | 28.26 | 23.12 | *hypogaea* | Virginia Bunch |
| ICG721 | H4 | H3 | . | . | . | . | H1 | H2 | H1 | H1 | H2 | . | H1 | . | 23.26 | 27.03 | 22.66 | *hypogaea* | Virginia Bunch |
| ICG5827 | . | . | . | H7 | . | H3 | H1 | H2 | H3 | H1 | H2 | H1 | H1 | H1 | 31.84 | 36.38 | 24.02 | *hypogaea* | Virginia Runner |
| ICG11219 | H4 | H3 | H2 | . | . | H3 | H1 | H2 | H1 | H1 | . | H1 | H1 | H1 | 22.78 | 33.83 | 23.59 | *hypogaea* | Virginia Runner |
| ICG3992 | H3* | . | . | H2 | H1 | . | H1 | . | H1 | H1 | . | . | H1 | . | 23.32 | 30.51 | 16.73 | *hypogaea* | Virginia Runner |
| ICG2773 | . | . | . | H2 | H1 | H3 | . | H2 | . | H1 | H2 | . | . | . | 20.2 | 29.87 | 25.23 | *hypogaea* | Virginia Runner |
| ICG2511 | H4 | H3 | H2 | H2 | . | H3 | H1 | H2 | . | H1 | H2 | H1 | H1 | H1 | 22.03 | 29.03 | 17.73 | *hypogaea* | Virginia Runner |
| ICG6813 | . | . | . | H2 | H1 | H3 | H1 | H2 | . | H1 | . | H1 | H1 | . | 19.46 | 26.94 | 18.5 | *hypogaea* | Virginia Runner |
| ICG4156 | H4 | H3 | H2 | . | . | . | H1 | H2 | H1 | . | . | H1 | H1 | H1 | 21.89 | 26.82 | 21.73 | *hypogaea* | Virginia Runner |

(*) Indicates superior haplotype

(-) Indicates haplotype not available
